# New furoisocoumarins and isocoumarins from the mangrove endophytic fungus *Aspergillus* sp. 085242

**DOI:** 10.3762/bjoc.12.196

**Published:** 2016-09-23

**Authors:** Ze’en Xiao, Senhua Chen, Runlin Cai, Shao’e Lin, Kui Hong, Zhigang She

**Affiliations:** 1 School of Chemistry and Chemical Engineering, Sun Yat-Sen University, No. 135 of Xingang West Road, Guangzhou, 510275, China; 2 Shenzhen Academy of Metrology and Quality Inspection, No. 144 of Minkan Road, Minzhi Street, Longhua District, Shenzhen, 518102, China; 3 Key Laboratory of Combinatorial Biosynthesis and Drug Discovery, Ministry of Education of China, School of Pharmaceutical Sciences, Wuhan University, Wuhan, 430071, China

**Keywords:** α-glucosidase, *Aspergillus*, DPPH^·^, furoisocoumarin, isocoumarin

## Abstract

The chemical investigation of the mangrove endophytic fungus *Aspergillus* sp. 085242 afforded eight isocoumarin derivatives **1**–**8** and one isoquinoline **9**. Asperisocoumarins A and B (**1** and **2**) were new furoisocoumarins, and asperisocoumarins E and F (**5** and **6**) were new isocoumarins. Their structures were established by analysis of their spectroscopic data and the absolute configuration of compound **2** was unambiguously determined by X-ray structure analysis and ECD calculation. Moreover, the absolute configurations of compounds **3**–**5** were assigned by comparison of their ECD spectra with isocoumarins described in the literature. Asperisocoumarins C and D (**3** and **4**) were fully characterized spectroscopically and isolated from a natural source for the first time. Asperisocoumarins A–D (**1**–**4**) related to the class of furo[3,2-*h*]isocoumarins are rarely occurring in natural sources. Compounds **2**, **5**, and **6** showed moderate α-glucosidase inhibitory activity with IC_50_ of 87.8, 52.3, and 95.6 μM, respectively. In addition, compounds **1** and **3** exhibited weak radical scavenging activity with EC_50_ values of 125 and 138 μM, respectively.

## Introduction

Isocoumarins are an important group of natural products with diverse structural features and interesting biological activities. They have been widely isolated from fungi, lichens, bacteria, plants, and insects [1–2]. Furoisocoumarins combining a furan ring and an isocoumarin moiety are divided into two subclasses depending on their fusion type: linear furo[2,3-*g*]isocoumarins and angular type furo[3,2-*h*]isocoumarins. The linear furo[2,3-*g*]isocoumarins are relatively common in nature and coriandrin [3–4], dihydrocoriandrin [3–4], coriandrone C [5], and coriandrone E [5] are a few examples. However, an angular-type furo[3,2-*h*]isocoumarin has up to date only been once reported from a natural source: Coriandrone A isolated from the aerial parts of *Coriandrum sativum* [6] and other furo[3,2-*h*]isocoumarins have been reported as synthetic products [7–9].

**Figure 1:**
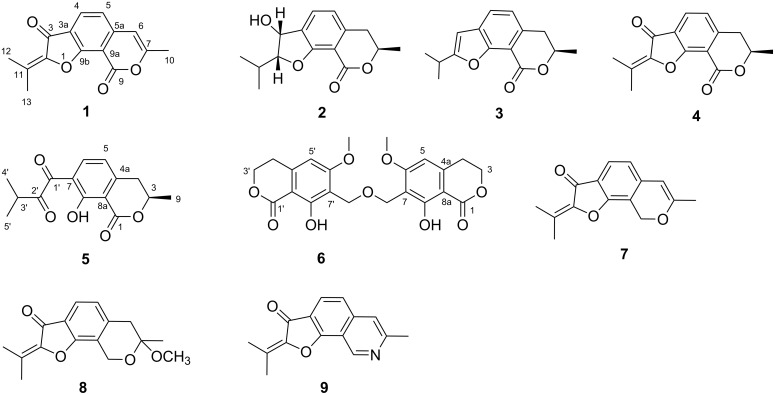
The structure of compounds **1**–**9**.

In the last decade, our research group has been devoted to finding novel bioactive compounds from mangrove endophytic fungi derived from the South China Sea [10–15]. In a previous study, a chemical investigation of the endophytic fungal strain *Aspergillus* sp. 085242 allowed us to identify two novel sesquiterpenoids, asperterpenols A and B with an unusual 5/8/6/6 tetracyclic ring skeleton [15]. The unique structures of these sesquiterpenoids encouraged us to further study this fungal strain and continuous chemical investigation of it led to the isolation of two new furo[3,2-*h*]isocoumarins, asperisocoumarins A and B (**1** and **2**) and two new isocoumarins, asperisocoumarins E and F (**5** and **6**), together with five known compounds (**3**, **4**, **7**–**9**) (Figure 1). Details of the isolation, structure elucidation, and biological activity of these compounds are reported herein.

## Results and Discussion

The mangrove endophytic fungus *Aspergillus* sp. 085242 was cultured on solid rice medium with saline water for four weeks. The MeOH extract of the fermentation was fractionated by repeated silica gel chromatography and Sephadex LH-20 column chromatography to yield compounds **1**−**9**.

**Figure 2:**
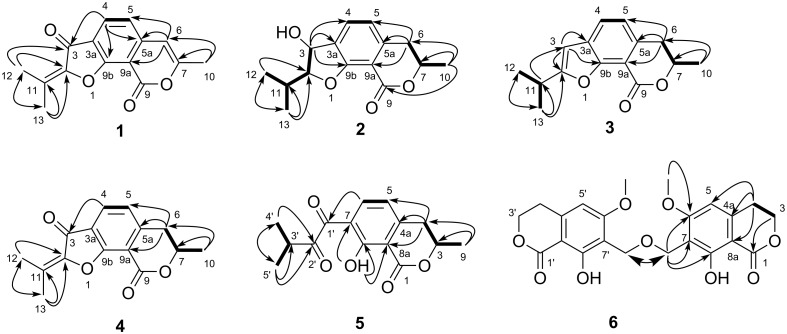
Key HMBC (arrows) and COSY (bold lines) correlations of compounds **1**−**6**.

Compound **1** was obtained as pale yellow crystal. Its molecular formula was established as C_15_H_12_O_4_ on the basis of HREIMS (*m*/*z* 256.0729, calcd for C_15_H_12_O_4_, 256.0730) and NMR data, implying ten degrees of unsaturation. The IR spectrum displayed intense absorption bands at 1737 and 1695 cm^−1^ indicating the presence of two carbonyl functionalities. The ^1^H NMR data (Table 1) showed two aromatic AB spin system protons δ_H_ 7.92 (1H, d, *J* = 8.0 Hz, H-4) and δ_H_ 7.01 (1H, d, *J* = 8.0 Hz, H-5), one olefinic proton δ_H_ 6.30 (1H, s, H-6), and three methyl groups δ_H_ 2.39 (3H, s, H-13), δ_H_ 2.32 (3H, s, H-10), and δ_H_ 2.24 (3H, s, H-12). ^13^C and DEPT NMR spectra of **1** revealed the resonance of two carbonyl, six aromatic, four olefinic, and three methyl carbons. Key HMBC correlations (Figure 2) from H-10 to C-6 and C-7, H-6 to C-5, C-5a, and C-9a, H-4 to C-3a, C-5a, and C-9b, and the upfield appearance of carbonyl group C-9 (δ_C_ 158.2) established a 3a,9b-disubstituted 7-methylisocoumarin unit. A 2-oxy-3-methyl-2-butenoyl moiety was assigned by the HMBC correlations of two methyl protons H-12 and H-13 to C-2, C-3, and C-11, as well as the chemical shifts of these carbons. This moiety connected to the aromatic ring at C-3a was supported by the HMBC correlations of H-4 with C-3. An ether linkage between C-2 and C-9b was fused as a 3-oxobenzofuran unit according to the chemical shifts of C-2 (δ_C_ 145.7) and C-9b (δ_C_ 165.1) as well as the required degrees of unsaturation. Thus, the structure of **1** was identified as 7-methyl-2-(propan-2-ylidene)-9*H*-furo[3,2-*h*]isochromene-3,9(2*H*)-dione, named asperisocoumarin A.

**Table 1:** NMR spectroscopic data of compounds **1**–**4**.^a^

No.	**1**		**2**		**3**		**4**	

	δ_C_	δ_H_ (*J* in Hz)	δ_C_	δ_H_ (*J* in Hz)	δ_C_	δ_H_ (*J* in Hz)	δ_C_	δ_H_ (*J* in Hz)

2	145.7, C		94.5, CH	4.17, dd (5.5, 10.2)	167.2, C		145.3, C	
3	181.6, C		71.0, CH	5.09, d (5.5)	99.6, CH	6.39, d (0.9)	182.3, C	
3a	123.1, C		131.2, C		130.1, C		124.6, C	
4	130.3, CH	7.92, d (8.0)	130.9, CH	7.53, d (7.5)	125.8, CH	7.61, d (6.6)	129.4, CH	7.88, d ( 7.7)
5	119.1, CH	7.01, d (8.0)	119.6, CH	6.76, d (7.5)	121.5, CH	7.03, d (6.6)	121.5, CH	7.01, d (7.7)
5a	145.7, C		142.3, C		135.3, C		148.4, C	
6	104.2, CH	6.30, s	35.3, CH_2_	2.90, d (8.4); 2.89, d (5.4)	35.3, CH_2_	3.01, m	36.0, CH_2_	3.02, m
7	159.0, C		74.6, CH	4.61, m	75.4, CH	4.70, m	74.7, CH	4.70, m
9	158.2, C		162.4, C		162.8, C		161.4, C	
9a	105.7, C		109.3, C		109.9, C		110.9, C	
9b	165.1, C		161.6, C		153.8, C		164.3, C	
10	20.1, CH_3_	2.32, s	20.8, CH_3_	1.43, d (6.3)	21.3, CH_3_	1.54, d (6.4)	20.8, CH_3_	1.55, d (6.3)
11	135.3, C		27.3, CH	2.31, m	28.3, CH	3.20, m	135.6, C	
12	20.8, CH_3_	2.24, s	20.0, CH_3_	1.29, d (6.6)	21.0, CH_3_	1.38, d (6.9)	20.9, CH_3_	2.22, s
13	17.7, CH_3_	2.39, s	19.4, CH_3_	1.14, d (6.6)	21.0, CH_3_	1.37, d (6.9)	17.8, CH_3_	2.39, s

^a1^H (400 MHz) and ^13^C (100 MHz) in CDCl_3_.

**Figure 3:**
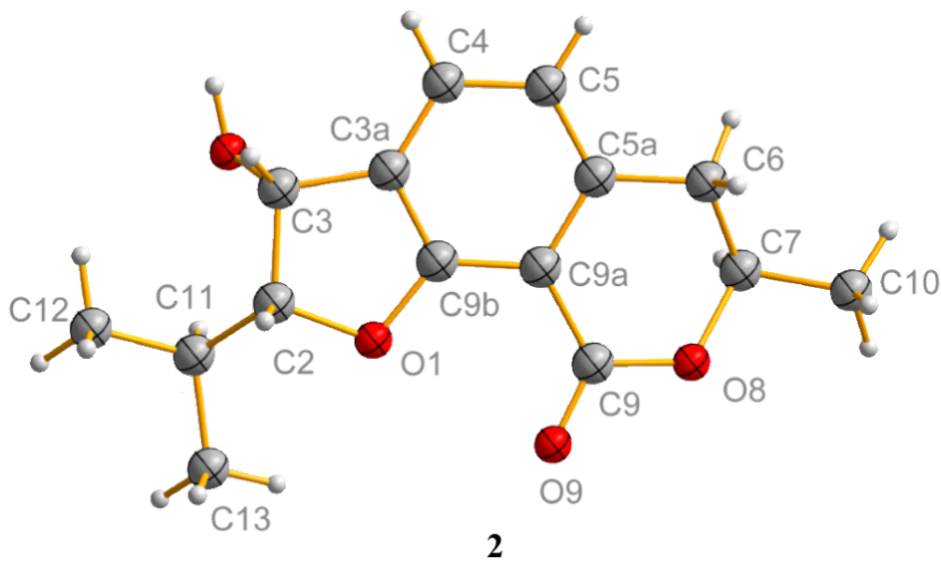
ORTEP structure of compound **2**.

**Figure 4:**
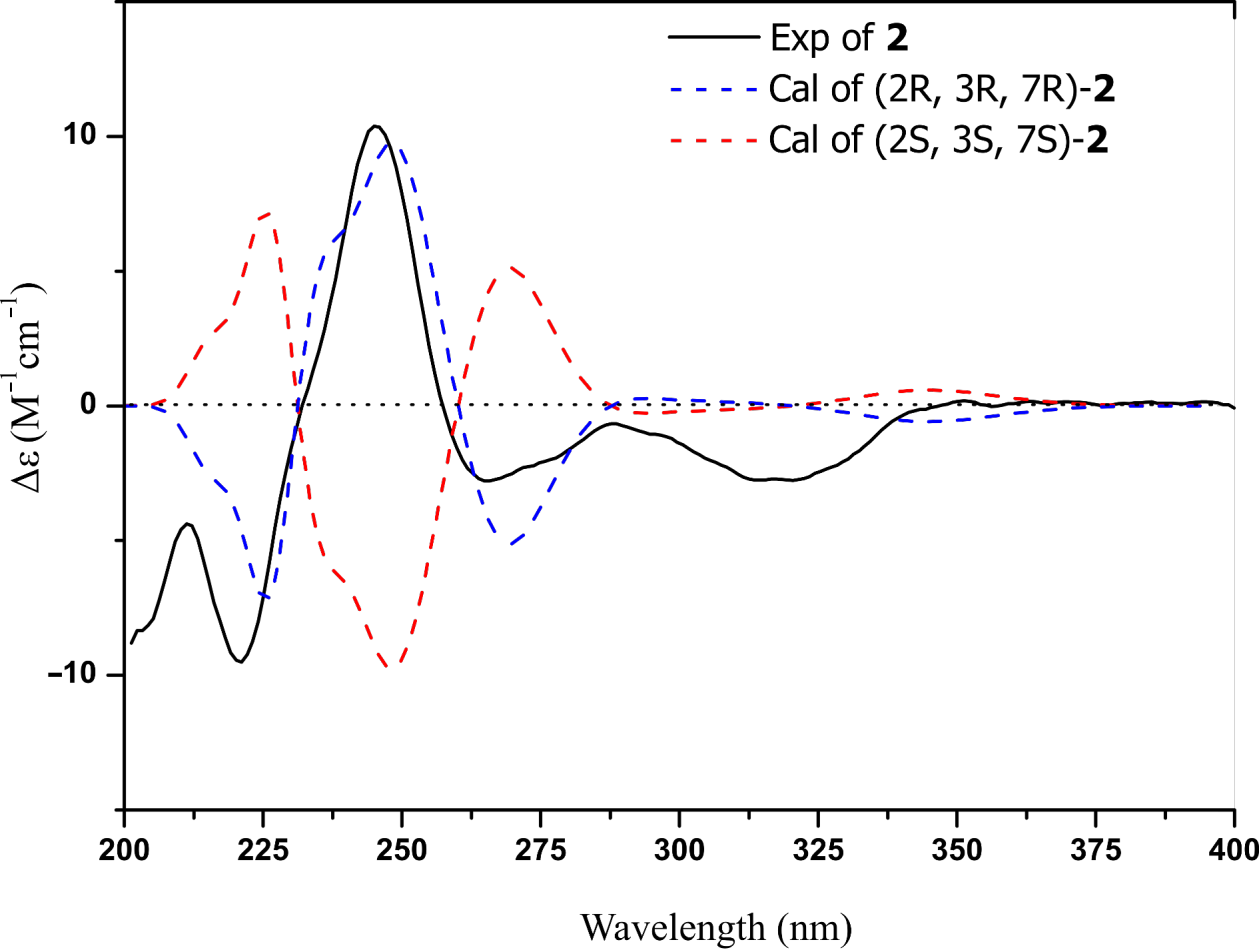
Comparison of the experimental and calculated ECD spectra of **2**.

Compound **2** was obtained as colorless crystals, and analyzed for the molecular formula C_15_H_18_O_4_ by interpretation of HREIMS (*m*/*z* 262.1201, calcd for C_15_H_18_O_4_, 262.1200). The IR spectrum revealed the presence of an additional hydroxy group at 3409 cm^−1^ and the absence of a carbonyl group at 1737 cm^−1^ in comparison with compound **1**. The ^1^H NMR spectrum (Table 1) showed the signals corresponding to two ortho-coupled aromatic protons, four alkyl methine protons, one methylene, and three methyl groups. The ^13^C NMR spectrum of **2** displayed the resonance of one carbonyl, six aromatic, four methine centers including three oxygenated, one methylene, and three methyl carbons. The above spectroscopic data suggested that compound **2** was a hexahydro-analogue of **1**. This deduction was further evidenced by the HMBC correlations of H-10 to C-9, C-7 and C-6, H-6 to C-5, C-5a and C-9a, and H-3 to C-3a, C-4 and C-9b, as well as the COSY correlations of H-2 with H-3 and H-11, H-11 with H-12 and H-13, H-4 with H-5, and H-7 with H-6 and H-10 (Figure 2). The relative configuration of compound **2** was determined by X-ray crystallographic analysis (Figure 3). The final refinement of the CuKα data resulted in a Flack parameter of 0.12(16) and the Hooft parameter of 0.06(8) [16–17], which allowed the assignment of the absolute configuration of **2** as (2*R*,3*R*,7*R*) (Figure 3). Moreover, the predicted ECD curves of **2** and its relevant enantiomer were computed at the [B3LYP/6-31 G(2d,p)] level, and the experimental ECD curve of **2** agreed well with the predicted one (Figure 4), in accordance with the deduction from the X-ray crystallography analysis. Therefore, the structure of compound **2** was established as depicted in Figure 3 and named asperisocoumarin B.

**Figure 5:**
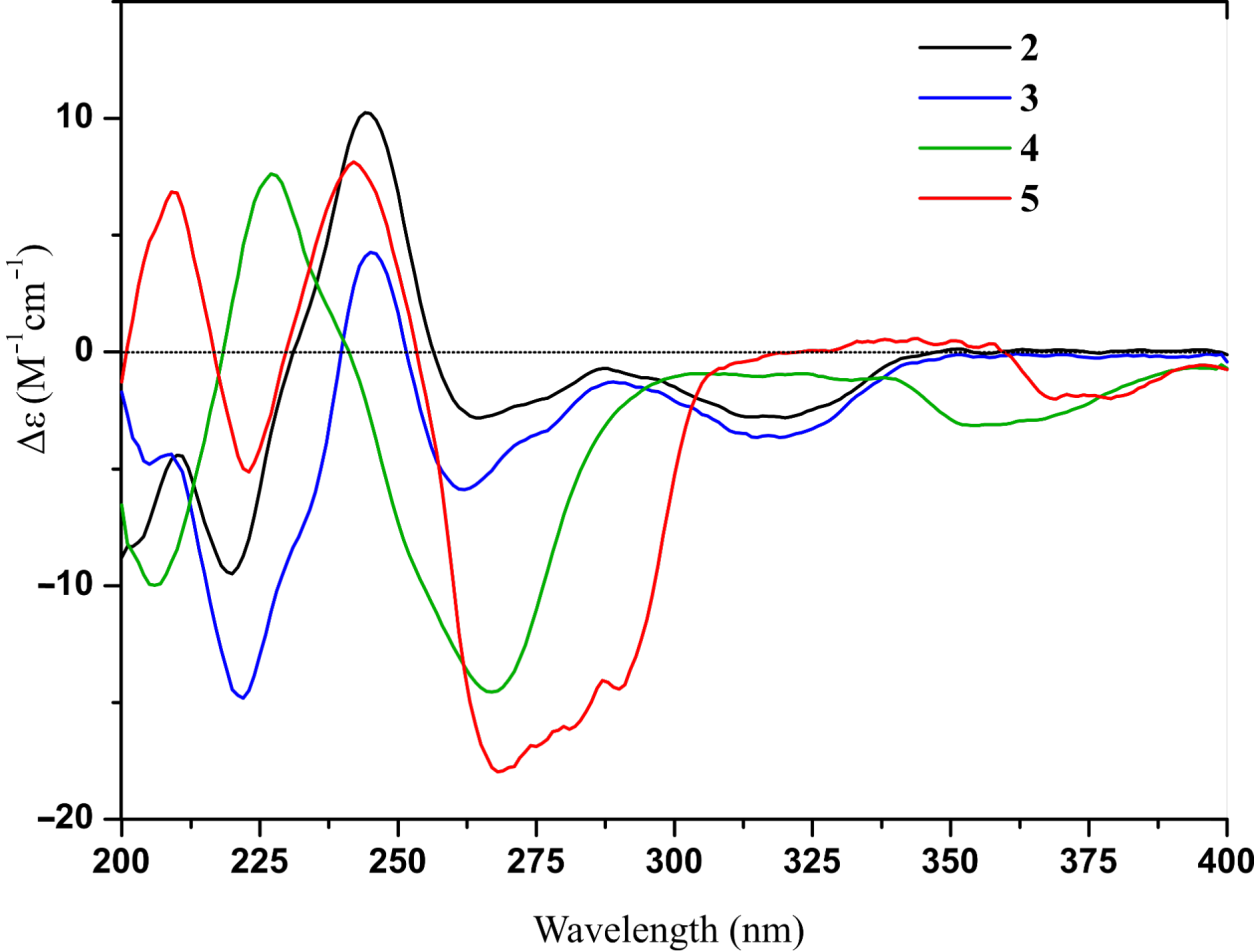
The experimental ECD spectra of **2**–**5**.

Asperisocoumarin C (**3**) was obtained as a pale yellow crystalline solid and displayed an [M + H]^+^ ion in HRESIMS at *m*/*z* 245.1174, suggesting a molecular formula of C_15_H_16_O_3_. A careful comparison of its ^1^H and ^13^C NMR spectra (Table 1) with those of **2** indicated that compound **3** also shared the same isocoumarin skeleton as compound **2**. The main differences were that an additional olefinic methine carbon at δ_C_ 99.6 and a quaternary carbon at δ_C_ 167.2 were observed, whereas two oxygenated methine carbons at δ_C_ 94.5 and 71.0 were absent in the spectrum of compound **3**. These differences were further supported by the HMBC correlations of olefinic proton H-3 to C-2, C-3a and C-9b (Figure 2). By comparison of the ECD spectrum (Figure 5) and the optical rotation of **3** with data reported for dihydrocoumarins [18], it was possible to assign the absolute configuration of C-7 as *R*. So, the structure of compound **3** was identified as (*R*)-2-isopropyl-7-methyl-6,7-dihydro-9*H*-furo[3,2-*h*]isochromen-9-one.

Asperisocoumarin D (**4**) was isolated as a white amorphous powder. Its molecular formula was determined as C_15_H_14_O_4_ by HREIMS (*m*/*z* 258.0888, calcd for C_15_H_14_O_4_, 258.0887). The ^1^H and ^13^C NMR spectra of **4** were similar to those of asperisocoumarin A (**1**), except that the NMR resonances assigned to olefinic carbons C-6 and C-7 were replaced by sp^3^ hybridized methylene (δ_C_ 36.0, δ_H_ 3.02) and methine (δ_C_ 74.7, δ_H_ 4.70) signals. HMBC correlations from H-10 (δ_H_ 2.12) to C-6 and C-7 and COSY correlations of H-7 with H-6 and H-10 further supported the above deduction (Figure 2). Finally, negative cotton effect (λ = 265 nm, Δε = −15.10) observed in the ECD spectrum (Figure 5), allowed the definition of the absolute configuration at C-7 (*R*) of compound **4** [18]. Thus, the structure of compound **4** was identified as (*R*)-2-isopropyl-7-methyl-6,7-dihydro-9*H*-furo[3,2-*h*]isochromen-9-one.

Asperisocoumarin E (**5**) was obtained as a pale yellow powder and the molecular formula was deduced by HREIMS analysis as C_15_H_16_O_5_ (*m*/*z* 276.0092, calcd for C_15_H_16_O_5_, 276.0092), indicating eight degrees of unsaturation. The ^1^H and ^13^C NMR spectra of compound **5** were quite similar to those of **3** except for absence of an olefinic proton at H-3 and presence of two additional carbonyl carbons at C-1' (δ_C_ 206.2) and C-2' (δ_C_ 193.9), respectively (Table 2). This evidence suggested that compound **5** lacks a furan ring and is most likely be derived from the furan ring-opening and oxidation of compound **3**, which was established by HMBC correlations of the aromatic proton H-6 to C-1', two methyl protons H-4' and H-5' to C-2', and chelated hydroxy proton 8-OH to C-8, C-7, and C-8a (Figure 2). The negative circular dichroism at 265 nm (Figure 5) suggested *R* configuration at C-3, by comparison with data for isocoumarin derivatives described in the literature [13]. Thus, asperisocoumarin E (**5**) was elucidated as (*R*)-1-(8-hydroxy-3-methyl-1-oxoisochroman-7-yl)-3-methylbutane-1,2-dione.

**Table 2:** NMR Spectroscopic data for **5** and **6**.^a^

No.	**5**		No.	**6**	
	
	δ_C_	δ_H_ (*J* in Hz)		δ_C_	δ_H_ (*J* in Hz)

1	169.4, C		1/1'	169.7, C	
3	76.0, CH	4.80, m	3/3'	67.7, CH_2_	4.51, t (6.1)
4	34.8, CH_2_	3.01, d (9.9), 3.03, d (5.0)	4/4'	28.3, CH_2_	3.01, t (6.1)
4a	146.4, C		4a/4a'	141.6, C	
5	118.8, CH	8.03, d (7.9)	5/5'	101.4, CH	6.27, s
6	137.0, CH	6.87, d (7.9)	6/6'	164.6, C	
7	121.9, C		7/7'	112.8, C	
8	162.4, C		8/8'	162.7, C	
8a	109.3, C		8a/8a'	102.4, C	
9	20.7, CH	1.58, d (6.3)	9/9'	60.1, CH_2_	4.67, s
1'	206.2, C		6/6'-OCH_3_	56.1, CH_3_	3.87, s
2'	193.9, C		8/8'-OH		11.31, s
3'	36.6, CH	3.18, m			
4'	17.2, CH_3_	1.29, d (7.0)			
5'	17.3, CH_3_	1.31, d (7.0)			
8-OH		11.94, s			

^a1^H (400 MHz) and ^13^C (100 MHz) in CDCl_3_.

Asperisocoumarin F (**6**) was obtained as a white powder. The molecular formula of **6** was deduced as C_22_H_22_O_9_ from HRESIMS analysis (*m*/*z* 429.1186 [M − H]^−^), implying 12 degrees of unsaturation. Its ^1^H NMR spectrum resembled that of stellatin [13], except the absence of a hydroxy proton at δ_H_ 2.24. In the ^13^C NMR spectrum, the chemical shift value of the oxygenated methylene at C-9 was 3.9 ppm higher than that of stellatin [19]. At the same time, there was a strong HMBC correlation between H-9 and C-9' or H-9' and C-9. The above evidences allowed us to conclude that compound **6** was a polyether dimer of stellatin as shown in Figure 2.

**Figure 6:**
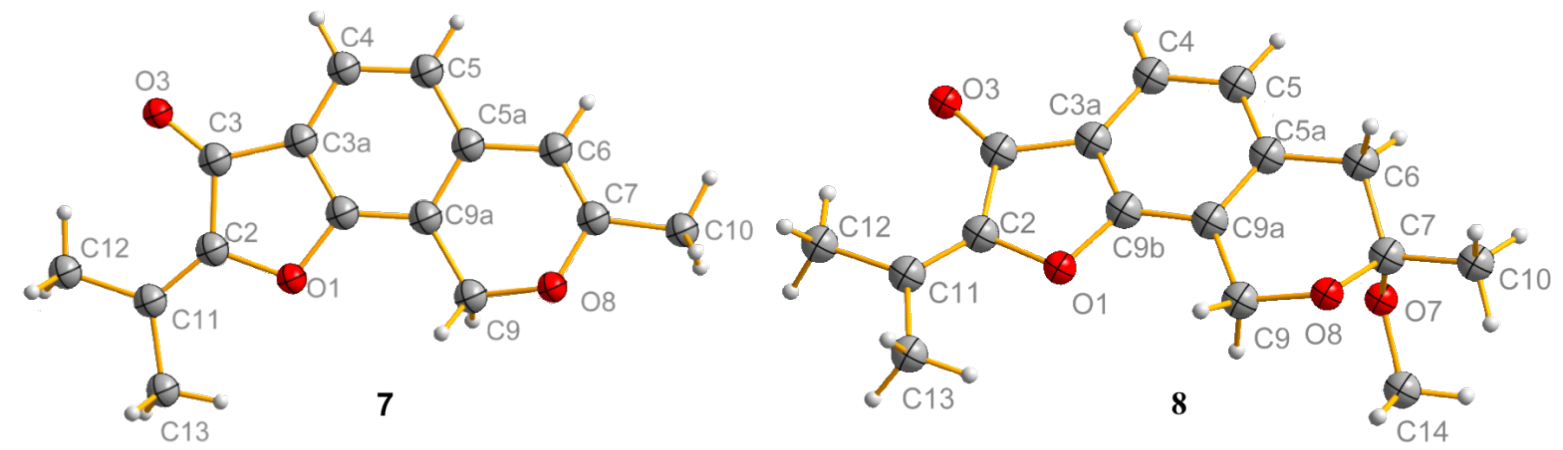
ORTEP structures of compound **7** and **8**.

In addition, the following known compounds were identified: ustusorane B (**7**) [20], penicisochroman A (**8**) [21], and TMC-120B (**9**) [22], on the basis of the spectroscopic comparison with those reported in the literature as well as to the specific rotation. The structures of ustusorane B (**7**) and penicisochroman A (**8**) were analyzed by X-ray crystallography analysis (Figure 6) for the first time.

Numerous isocoumarin and 3,4-dihydroisocoumarin derivatives have been isolated from various natural sources [1]. However, the furo[3,2-*h*]isocoumarin is a very uncommon class of isocoumarins and to date only a few members have been reported [1,6]. Asperisocoumarin A–D (**1**–**4**) were the second examples belonging to furo[3,2-*h*]isocoumarins from natural sources. Asperisocoumarin B (**2**) shared the same planar structure as (2*S*,3*S*,7*R*)-6,9-dihydro-3-hydroxy-7-methyl-2-(1-methylethyl)-7*H*-furo[3,2-*h*][2]benzopyran-9(2*H*)-one, which was the intermediate of the syntheses of (−)-ustusorane D and (+)-penicisochroman B [8]. Asperisocoumarin C (**3**) and D (**4**) had the same structure as (*R*)-2-isopropyl-7-methyl-6,7-dihydro-9*H*-furo[3,2-*h*]isochromen-9-one and (*R*)-7-methyl-2-(propan-2-ylidene)-6,7-dihydro-9*H*-furo[3,2-*h*]isochromene-3,9-(2*H*)-dione, respectively. Both have been synthesized as intermediates during synthesis and structural characterization of natural benzofuranoids [9]. Asperisocoumarin E (**5**) containing an isopentenyl substituent with two adjacent carbonyl groups seems to be rare in natural isocoumarin derivatives and asperisocoumarin F (**6**) presents as a scaffold with an ether dimer of isocoumarin.

All isolates were evaluated for their α-glucosidase inhibitory activity using clinical acarbose (IC_50_ of 628.3 μM) as a positive control. Compounds **2**, **5**, and **6** showed moderate α-glucosidase inhibitory activity with IC_50_ of 87.8, 52.3, and 95.6 μM, respectively. The other compounds were inactive (> 300 μM). Compounds **1**–**9** were also evaluated for antibacterial activities against *Staphylococcus aureus*, *Staphylococcus epidermidis*, *Escherichia coli*, *Klebsiella pneumoniae*, and *Bacillus subtilis.* None of the compounds was active at a concentration of 50 μg/mL. In the free radical scavenging assay using 2,2-diphenyl-1-picrylhydrazyl (DPPH), only compounds **1** and **3** exhibited weak activity with EC_50_ values of 125 and 130 μM, respectively (vitamin C was used as a positive control with EC_50_ = 35 μM).

## Experimental

**General experimental procedures.** Analogously as described in reference [12]. Melting points were determined with a Fisher-Johns hot-stage apparatus apparatus and are uncorrected. UV data were measured on a PERSEE TU-1900 spectrophotometer. Infrared spectra were recorded on a Nicolet Nexus 670 spectrophotometer using KBr discs. EIMS data were measured on a DSQ EI-mass spectrometer (Thermo, Shanghai, China) and HREIMS data were carried out on a DMAT95XP high-resolution mass spectrometer. ESIMS spectra were recorded on a Finnigan LCQ-DECA mass spectrometer and HRESIMS spectra were determined on a Shimadzu LCMS-IT-TOF mass spectrometer. 1D and 2D NMR spectra were carried out on Bruker Avance 400 spectrometer (^1^H 400 MHz, ^13^C 100 MHz). Chemical shifts (δ) are given in ppm with reference to the solvent signal (δ_C_ 77.1/δ_H_ 7.26 for CDCl_3_) and coupling constants (*J*) are given in Hz. ECD spectra were measured on a Chirascan CD spectrometer (Applied Photophysics, London, UK). Single-crystal data were collected on an Agilent Gemini Ultra diffractometer (CuKα radiation). Silica gel (200–300 mesh, Qingdao Marine Chemical Factory) and Sephadex LH-20 (Amersham Pharmacia, Piscataway) were used for column chromatography (CC). Thin layer chromatography was performed on precoated silica gel plates (Qingdao Huang Hai Chemical Group Co., G60, F-254).

**Fungal material.** The fungus *Aspergillus* sp. 085242 was isolated from healthy roots of *Acanthus ilicifolius*, which were collected from the Shankou Mangrove National Nature Reserve in Guangxi Province, China. Fungal identification was carried out using a molecular biological protocol by DNA amplification and sequencing of the ITS region and the sequence data have been submitted to GenBank with accession no. KC816018.1. A BLAST search result indicated that the sequence was the most similar (99%) to the sequence of *Aspergillus* sp. (compared to KP059102.1 and KJ567455.1)*.* A voucher strain is deposited in the China Center for Type Culture Collection under patent depository number CCTCC M 2013081.

**Fermentation, extraction, and isolation.** Analogously as described in reference [12] the fungus was grown on autoclaved rice solid substrate medium (thirty 500 mL Erlenmeyer flasks, each containing 50 g rice and 50 mL 3‰ of saline water) at room temperature under static conditions and daylight for 28 days. Following incubation, the mycelia and solid rice medium were extracted with MeOH three times. The extract was evaporated under reduced pressure to yield 41 g of residue. The residue was then divided into 20 fractions (Fr. 1–Fr. 20) by column chromatography on silica gel eluted by a gradient of petroleum ether/EtOAc from 1:0 to 0:1. Fr. 4 (309 mg) was applied to the Sephadex LH-20 CC (CHCl_3_/MeOH, v/v, 1:1) to give subfraction Fr. 4.9, which was purified on silica gel (petroleum ether/EtOAc, v/v, 8:2) to yield **1** (2.8 mg). Fr. 6 (105 mg) was rechromatographed on silica gel (petroleum ether/EtOAc, v/v, 8:2) to give subfraction Fr. 6.8, which was puriﬁed by Sephadex LH-20 CC (CHCl_3_/MeOH, v/v, 1:1) to yield **7** (2.3 mg) and **8** (2.1 mg). Fr. 7 (264 mg) was subsequently separated by Sephadex LH-20 CC eluted with (CHCl_3_/MeOH, v/v, 1:1) to give subfraction Fr. 7.9, which was purified on silica gel (petroleum ether/EtOAc, v/v, 7:3) to yield **2** (5.4 mg) and **3** (2.5 mg), respectively. Fr. 9 was chromatographed on Sephadex LH-20 CC (CHCl_3_/MeOH, v/v, 1:1) to give subfraction Fr. 9.7, which was puriﬁed using silica gel (petroleum ether/EtOAc, v/v, 7:3) to give **4** (4.1 mg) and **5** (2.6 mg). Fr. 11 (180 mg) was subsequently separated by Sephadex LH-20 CC eluted with MeOH to obtain **6** (3.1 mg). Fr. 12 was chromatographed on silica gel (petroleum ether/EtOAc, v/v, 6:4) to produce **9** (5.2 mg).

Asperisocoumarin A (**1**): pale yellow crystals; mp 189.5–192.0 °C; UV (MeOH) λ_max_ (log ε): 240 (4.25), 357 (3.56) nm; IR (KBr) ν_max_: 3074, 2998, 2904, 1737, 1695, 1656, 1606, 1576, 1458, 1343, 1265, 1164, 1078 cm^−1^; EIMS (*m*/*z*): 256; HRMS–EI (*m*/*z*): C_15_H_12_O_4_ calcd for 256.0730; found, 256.0729; ^1^H NMR (CDCl_3_, 400 MHz) and ^13^C NMR (CDCl_3_, 100 MHz), see Table 1.

Asperisocoumarin B (**2**): colorless crystals; mp 179.4–181.4 °C; [α]_D_^20^ +25.3 (*c* 0.02, MeOH); UV (MeOH) λ_max_ (log ε): 220 (4.83), 248 (4.25), 317 (3.56) nm; IR (KBr) ν_max_: 3391, 2973, 2935, 2868, 1691, 1610, 1451, 1383, 1175, 1054 cm^−1^; EIMS (*m*/*z*): 262; HRMS–EI (*m*/*z*): C_15_H_18_O_4_ calcd for 262.1200; found, 262.1201; ^1^H NMR (CDCl_3_, 400 MHz) and ^13^C NMR (CDCl_3_, 100 MHz), see Table 1.

Asperisocoumarin C (**3**): pale yellow amorphous powder; mp 122.4–124.8 °C; [α]_D_^20^ −78.9 (*c* 0.02, MeOH); UV (MeOH) λ_max_ (log ε): 221 (4.85), 252 (4.26), 324 (3.54) nm; IR (KBr) ν_max_: 3391, 2973, 2935, 2868, 1691, 1610, 1451, 1383, 1175, 1054 cm^−1^; EIMS (*m*/*z*) 244; HRMS–ESI (*m*/*z*): [M + H]^+^ calcd for C_15_H_16_O_3_, 245.1173; found, 245.1174; ^1^H NMR (CDCl_3_, 400 MHz) and ^13^C NMR (CDCl_3_, 100 MHz), see Table 1.

Asperisocoumarin D (**4**): white amorphous powder; mp 182.2–184.4 °C; [α]_D_^20^ −70 (*c* 0.01, MeOH); UV (MeOH) λ_max_ (log ε): 222 (4.89), 249 (4.28), 319 (3.50) nm; IR (KBr) ν_max_: 3390, 2976, 2934, 2866, 1692, 1612, 1453, 1381, 1176, 1056 cm^−1^; EIMS (*m*/*z*): 258; HRMS–EI (*m*/*z*): C_15_H_14_O_4_ calcd for 258.0887; found, 258.0888; ^1^H NMR (CDCl_3_, 400 MHz), see Table 1.

Asperisocoumarin E (**5**): pale yellow crystals; mp 136.1–138.0 °C; [α]_D_^20^ −78.6 (*c* 0.02, MeOH); UV (MeOH) λ_max_ (log ε): 220 (4.84), 335 (3.56) nm; IR (KBr) ν_max_: 3376, 2979, 1707, 1607, 1431, 1389, 1273, 1135, 806 cm^−1^; EIMS (*m*/*z*): 276; HRMS–EI (*m*/*z*): C_15_H_16_O_5_ calcd for 276.0092; found, 276.0092; (^1^H NMR (CDCl_3_, 400 MHz) and ^13^C NMR (CDCl_3_, 100 MHz), see Table 2.

Asperisocoumarin F (**6**): white amorphous powder; mp 201.1–203.0 °C; UV (MeOH) λ_max_ (log ε): 268 (4.64), 299 (3.36) nm; IR (KBr) ν_max_: 3476, 2998, 1670, 1581, 1369, 1283, 1125 cm^−1^; EIMS (*m*/*z*): 430; HRMS–ESI (*m*/*z*): [M − H]^−^ calcd for C_15_H_18_O_4_, 429.1186; found, 429.1186; ^1^H NMR (CDCl_3_, 400 MHz) and ^13^C NMR (CDCl_3_, 100 MHz), see Table 2.

**X-ray crystallographic analysis.** Single crystal X-ray diffraction data were collected at 123 K on an Agilent Gemini Ultra diffractometer with CuKα radiation (λ = 1.54178 Å). The structures were solved by direct methods (SHELXS-97) and refined using full-matrix least-squares difference Fourier techniques. Hydrogen atoms bonded to carbons were placed on the geometrically ideal positions by the “ride on” method. Hydrogen atoms bonded to oxygen were located by the difference Fourier method and were included in the calculation of structure factors with isotropic temperature factors. Crystallographic data for **2**, **7** and **8** have been deposited with the Cambridge Crystallographic Data Centre. Copies of the data can be obtained, free of charge, on application to the Director, CCDC, 12 Union Road, Cambridge CB2 1EZ, UK (fax: 44-(0)1223-336033, or email: deposit@ccdc.cam.ac.uk).

Crystal data of **2**: C_15_H_18_O_4_, *M*r = 262.29, monoclinic, *a* = 16.4513(3) Å, *b* = 11.2236(2) Å, *c* = 8.27930(10) Å, α = 90.00, β = 116.344, γ = 90.00, *V* = 1369.95(4) Å^3^, space group *C*2, *Z* = 4, *D*_calcd_ = 1.272 mg/m^3^, μ = 0.754 mm^−1^, and *F*(000) = 560. Crystal dimensions: 0.44 × 0.41 × 0.40 mm^3^. Independent reflections: 1987 (*R*_int_ = 0.0180). The final *R*_1_ values were 0.0237, ω*R*_2_ = 0.0614 (I > 2σ(I)). The goodness of fit on *F*^2^ was 1.078. Flack parameter = 0.12(16). CCDC number: 1458037.

Crystal data of **7**: C_15_H_14_O_3_, *M*r = 242.26, monoclinic, *a* = 7.6380(3) Å, *b* = 13.8996(6) Å, *c* = 11.22829(4) Å, α = 90.00, β = 95.368(4), γ = 90.00, *V* = 1192.60(8) Å^3^, space group *P*2_1_/*n*, *Z* = 4, *D*_calcd_ = 1.349 mg/m^3^, μ = 0.093 mm^−1^, and *F*(000) = 512. Crystal dimensions: 0.42 × 0.33 × 0.23 mm^3^. Independent reflections: 2569 (*R*_int_ = 0.0228). The final *R*_1_ values were 0.0419, ω*R*_2_ = 0.0961 (I > 2σ(I)). The goodness of fit on *F*^2^ was 1.037. Flack parameter = 0.09(10). CCDC number: 1458039.

Crystal data of **8**: C_32_H_36_O_8_, *M*r = 548.60, triclinic, *a* = 6.5460(2) Å, *b* = 8.6973(3) Å, *c* = 12.0611(4) Å, α = 88.934(3), β = 85.192(3), γ = 85.357(3), *V* = 1369.95(4) Å^3^, space group *P*-1, *Z* = 2, *D*_calcd_ = 1.336 mg/m^3^, μ = 0.782 mm^−1^, and *F*(000) = 296. Crystal dimensions: 0.42 × 0.28 × 0.23 mm^3^. Independent reflections: 2423 (*R*_int_ = 0.0263). The final *R*_1_ values were 0.0350, ω*R*_2_ = 0.0899 (I > 2σ(I)). The goodness of fit on *F*^2^ was 1.067. CCDC number: 1458040.

**Calculation of ECD spectra**. Molecular Merck force field (MMFF) and DFT/TD-DFT calculations were carried out with Spartan’ 14 software (Wavefunction Inc., Irvine, CA, USA) and Gaussian 09 program, respectively. Conformers within 10 kcal/mol energy window were generated and optimized using DFT calculations at B3LYP/6-31G(d) level. Conformers with Boltzmann distribution over 1% were chosen for ECD calculations in methanol at B3lYP/6-311+g(2d,p) level. The IEF-PCM solvent model for MeOH was used. ECD spectra were generated using the program SpecDis 3.0 (University of Würzburg, Würzburg, Germany) and OriginPro 8.5 (OriginLab, Ltd., Northampton, MA, USA) from dipole-length rotational strengths by applying Gaussian band shapes with sigma = 0.30 eV and UV shift = +21 nm. All calculations were performed with High-Performance Grid Computing Platform of Sun Yat-Sen University.

**Biological assays.** The assays for antibacterial [23] and α-glucosidase inhibitory [23] were carried out as described previously.

The assay for DPPH radical scavenging activity was measured by a reported method [24–25], with slight modifications. Firstly, 180 μL of DPPH^·^ (150 μM in MeOH) and 20 μL of a series of test compound solutions (31.2, 62.5, 125, 250, 500 μM in MeOH) were mixed in each well of a 96-well microtiter plate. The reaction was measured by determination of the absorbance A_sample+DPPH_· using a microtiter plate reader at 490 nm after shaking for 30 min at room temperature in the dark. Twenty μL test samples of each concentration with 180 μL of MeOH were used as the blank measurement for each tested compound, and the absorbance was recorded as A_sample_. The absorbance of the mixture of 20 μL of MeOH and 180 μL of DPPH^·^ was recorded as A_DPPH_·, and the absorbance of the 200 μL of MeOH was measured as A_blank_. The natural antioxidant vitamin C was used as a positive control. Calculations of the DPPH^·^ scavenging activity was performed according to the following equation: scavenging activity (%) = [1 − (A_sample+DPPH_· − A_sample_)/(A_DPPH_· − A_blank_)] × 100%. All measurements were done in triplicate from two independent experiments. The reported EC_50_ was the average value of two independent experiments.

## Supporting Information

File 1 1D and 2D NMR, HREIMS, and HRESIMS spectra of the new compounds.
