# Supplementary material for: New furoisocoumarins and isocoumarins from the mangrove endophytic fungus Aspergillus sp. 085242
Source: Beilstein J Org Chem. 2016 Sep 23;12:2077–85. doi: 10.3762/bjoc.12.196 (PMC5082600; doi:10.3762/bjoc.12.196)
Supplement: File 1 — 1D and 2D NMR, HREIMS, and HRESIMS spectra of the new compounds. [file Beilstein_J_Org_Chem-12-2077-s001.pdf]

Supporting Information

for

New furoisocoumarins and isocoumarins from the mangrove endophytic fungus *Aspergillus* sp. 085242

Ze'en Xiao[1,2,§], Senhua Chen[1,§], Runlin Cai[1], Shao'e Lin[1], Kui Hong*[,3], and Zhigang She*[,1]

Address: [1]School of Chemistry and Chemical Engineering, Sun Yat-Sen University, No. 135 of Xingang West Road, Guangzhou, 510275, China, [2]Shenzhen Academy of Metrology and Quality Inspection, No. 144 of Minkan Road, Minzhi Street, Longhua District, Shenzhen, 518102, China and [3]Key Laboratory of Combinatorial Biosynthesis and Drug Discovery, Ministry of Education of China, School of Pharmaceutical Sciences, Wuhan University, Wuhan, 430071, China

Email: Kui Hong - kuihong31@gmail.com; Zhigang She - cesshzhg@mail.sysu.edu.cn

*Corresponding author

[§]Equally contributing authors

1D and 2D NMR, HREIMS, and HRESIMS spectra of the new compounds

Computational details S3
Figure S1 B3LYP/6-31G(d) optimized low-energy conformers of **2**..........................S4
Figure S2 Comparison of the experimental and calculated ECD spectra of **2**............S5
Figure S3 HREIMS spectrum of **1**...S6
Figure S4 ^{1}H NMR spectrum of **1** in $CDCl_3$...S6
Figure S5 ^{13}C NMR spectrum of **1** in $CDCl_3$..S7
Figure S6 ^{1}H–^{1}H COSY spectrum of **1** in $CDCl_3$...S7
Figure S7 HSQC spectrum of **1** in $CDCl_3$ S8
Figure S8 HMBC spectrum of **1** in $CDCl_3$ S8
Figure S9 HREIMS spectrum of **2**...S9
Figure S10 ^{1}H NMR spectrum of **2** in $CDCl_3$...S9
Figure S11 ^{13}C NMR spectrum of **2** in $CDCl_3$...S10
Figure S12 ^{1}H–^{1}H COSY spectrum of **2** in $CDCl_3$..S10
Figure S13 HSQC spectrum of **2** in $CDCl_3$ S11
Figure S14 HMBC spectrum of **2** in $CDCl_3$ S11
Figure S15 HREIMS spectrum of **3**...S12
Figure S16 ^{1}H NMR spectrum of **3** in $CDCl_3$...S12
Figure S17 ^{13}C NMR spectrum of **3** in $CDCl_3$...S13
Figure S18 HSQC spectrum of **3** in $CDCl_3$ S13
Figure S19 HMBC spectrum of **3** in $CDCl_3$ S14
Figure S20 HREIMS spectrum of **4**...S14
Figure S21 ^{1}H NMR spectrum of **4** in $CDCl_3$...S15
Figure S22 ^{13}C NMR spectrum of **4** in $CDCl_3$...S15
Figure S23 ^{1}H–^{1}H COSY spectrum of **4** in $CDCl_3$..S16
Figure S24 HSQC spectrum of **4** in $CDCl_3$ S16
Figure S25 HMBC spectrum of **4** in $CDCl_3$ S17
Figure S26 HRESIMS spectrum of **5**...S17
Figure S27 ^{1}H NMR spectrum of **5** in $CDCl_3$...S18
Figure S28 ^{13}C NMR spectrum of **5** in $CDCl_3$...S18
Figure S29 ^{1}H–^{1}H COSY spectrum of **5** in $CDCl_3$..S19
Figure S30 HSQC spectrum of **5** in $CDCl_3$ S19
Figure S31 HMBC spectrum of **5** in $CDCl_3$ S20
Figure S32 HRESIMS spectrum of **6**...S20
Figure S33 ^{1}H NMR spectrum of **6** in $CDCl_3$...S21
Figure S34 ^{13}C NMR spectrum of **6** in $CDCl_3$...S21
Figure S35 ^{1}H–^{1}H COSY spectrum of **6** in $CDCl_3$..S22
Figure S36 HSQC spectrum of **6** in $CDCl_3$ S22
Figure S37 HMBC spectrum of **6** in $CDCl_3$ S23

Computational details

Molecular Merck force field (MMFF) and DFT/TD-DFT calculations were carried out with Spartan' 14 software (Wavefunction Inc., Irvine, CA, USA) and Gaussian 09 program, respectively. Conformers within 10 kcal/mol energy window were generated and optimized using DFT calculations at B3LYP/6-31G(d) level. Conformers with Boltzmann distribution over 1% were chosen for ECD calculations in methanol at B3lYP/6-311+g(2d,p) level. The IEF-PCM solvent model for MeOH was used. ECD spectra were generated using the program SpecDis 3.0 (University of Würzburg, Würzburg, Germany) and OriginPro 8.5 (OriginLab, Ltd., Northampton, MA, USA) from dipole-length rotational strengths by applying Gaussian band shapes with sigma = 0.30 eV and UV shift = +21 nm. All calculations were performed with High-Performance Grid Computing Platform of Sun Yat-Sen University.

Result

Table S1: Energy Analysis for the Conformers of (2*R*,3*R*,7*R*)-**2**.

compound	Conformation	G (Hartree)	G (Kcal/mol)	ΔG (Kcal/mol)	Boltzmann Dist (%)
(2*R*,3*R*,7*R*)-**2**	**2a**	−883.632283748	−554481.8206	0.0000	87.18
	**2b**	−883.629991994	−554480.3825	1.4380	7.68
	**2c**	−883.628769196	−554479.6152	2.2053	2.10
	**2d**	−883.629048220	−554479.7903	2.0303	2.83

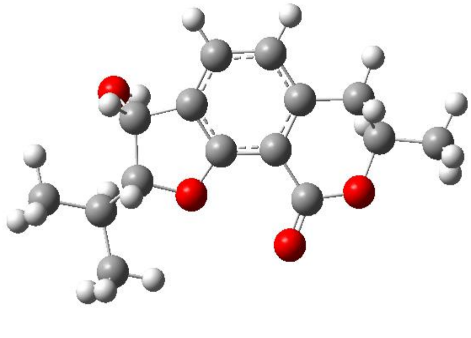

2a

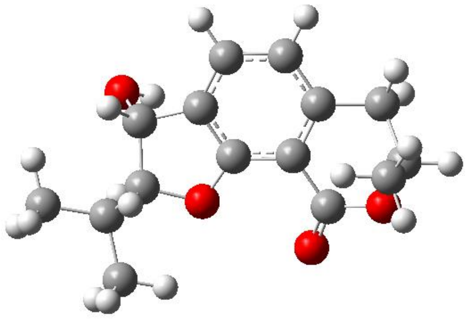

2b

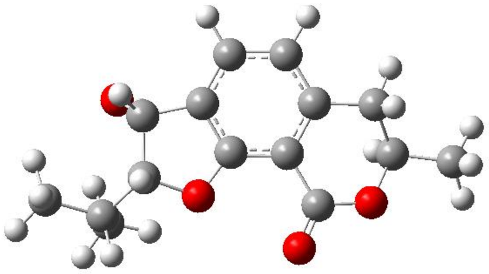

2c

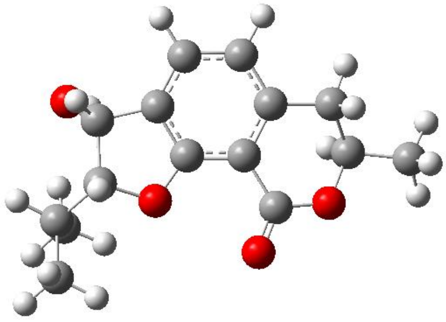

2d

Figure S1: B3LYP/6-31G(d) optimized low-energy conformers of **2**

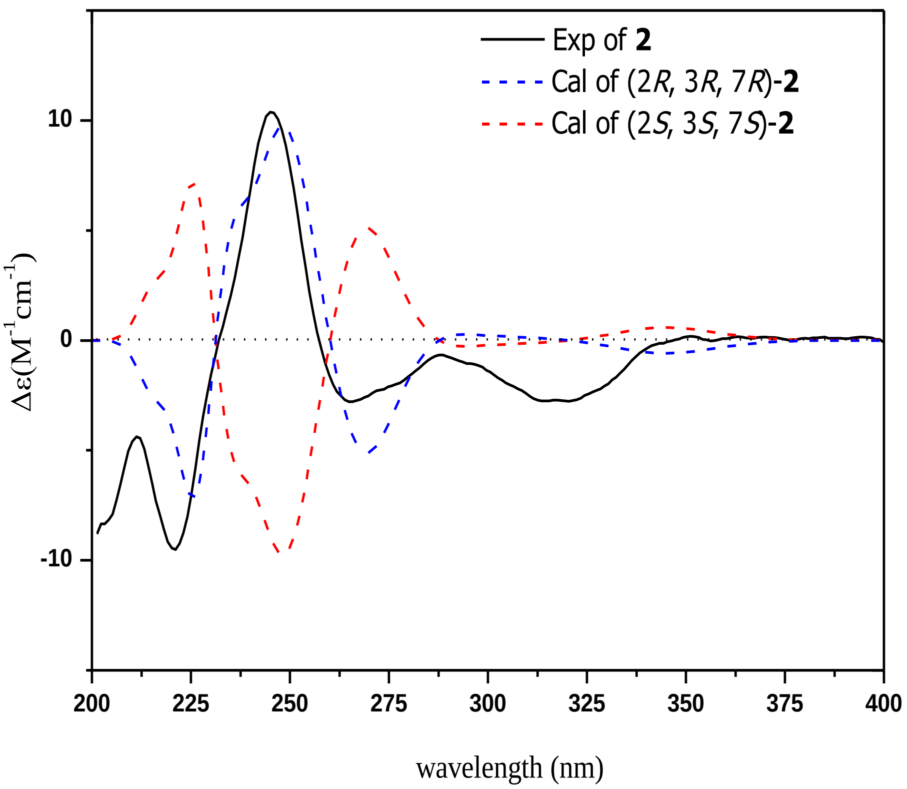

Figure S2: Comparison of the experimental ECD spectra of **2** with the B3lYP/6-311+g(2d,p) calculated spectrum of (2*R*,3*R*,7*R*)-**2** and (2*S*,3*S*,7*S*)-**2** in MeOH. σ = 0.30 eV.

References

Frisch, M. J.; Trucks, G. W.; Schlegel, H. B.; Scuseria, G. E.; Robb, M. A.; Cheeseman, J. R.; Scalmani, G.; Barone, V.; Mennucci, B.; Petersson, G. A.; Nakatsuji, H.; Caricato, M.; Li, X.; Hratchian, H. P.; Izmaylov, A. F.; Bloino, J.; Zheng, G.; Sonnenberg, J. L.; Hada, M.; Ehara, M.; Toyota, K.; Fukuda, R.; Hasegawa, J.; Ishida, M.; Nakajima, T.; Honda, Y.; Kitao, O.; Nakai, H.; Vreven, T.; Montgomery, J. A.; Peralta, J. E.; Ogliaro, F.; Bearpark, M.; Heyd, J. J.; Brothers, E.; Kudin, K. N.; Staroverov, V. N.; Keith, T.; Kobayashi, R.; Normand, J.; Raghavachari, K.; Rendell, A.; Burant, J. C.; Iyengar, S. S.; Tomasi, J.; Cossi, M.; Rega, N.; Millam, J. M.; Klene, M.; Knox, J. E.; Cross, J. B.; Bakken, V.; Adamo, C.; Jaramillo, J.; Gomperts, R.; Stratmann, R. E.; Yazyev, O.; Austin, A. J.; Cammi, R.; Pomelli, C.; Ochterski, J. W.; Martin, R. L.; Morokuma, K.; Zakrzewski, V. G.; Voth, G. A.; Salvador, P.; Dannenberg, J. J.; Dapprich, S.; Daniels, A. D.; Farkas, O.; Foresman, J. B.; Ortiz, J. V.; Cioslowski, J.; Fox, D. J. Gaussian 09, revision C.01. Gaussian, Inc.: Wallingford CT, 2010.Bruhn, T.; Schaumlöffel, A.; Hemberger, Y.; Bringmann, G. SpecDis: Quantifying the comparison of calculated and experimental electronic circular dichroism spectra. Chirality 2013, 25, 243–249.

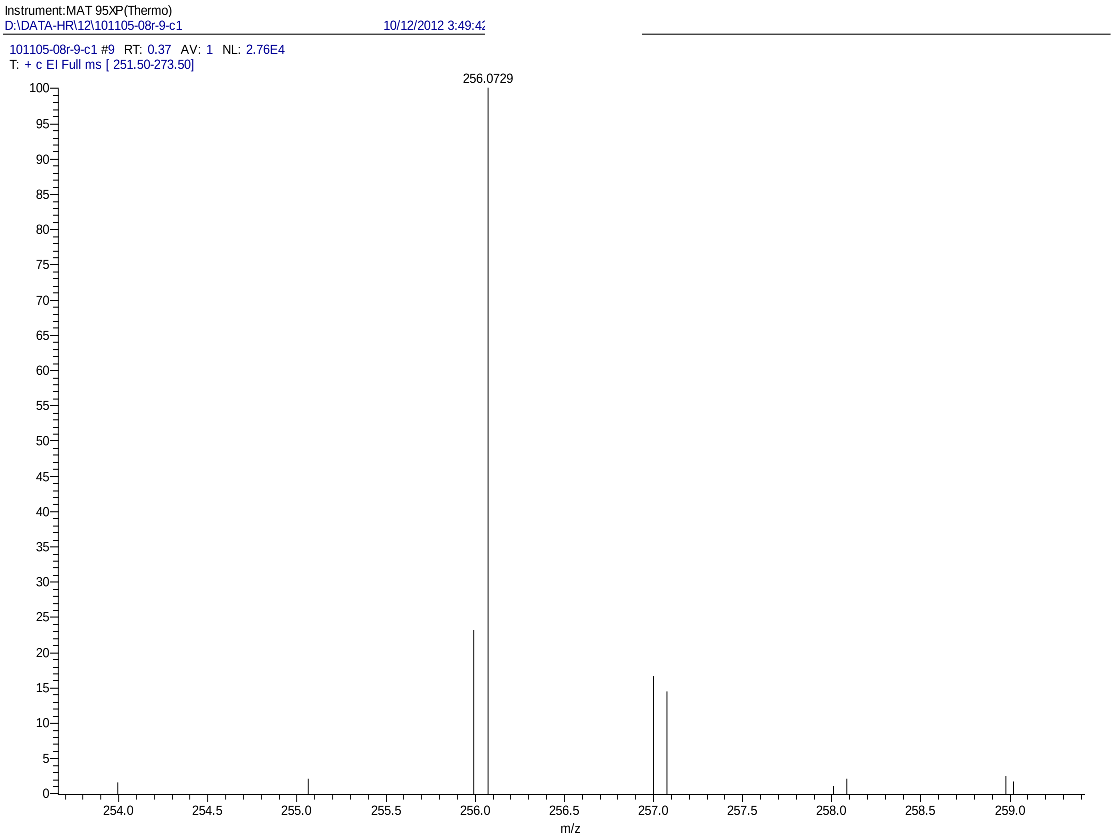

Figure S3 HREIMS spectrum of **1**

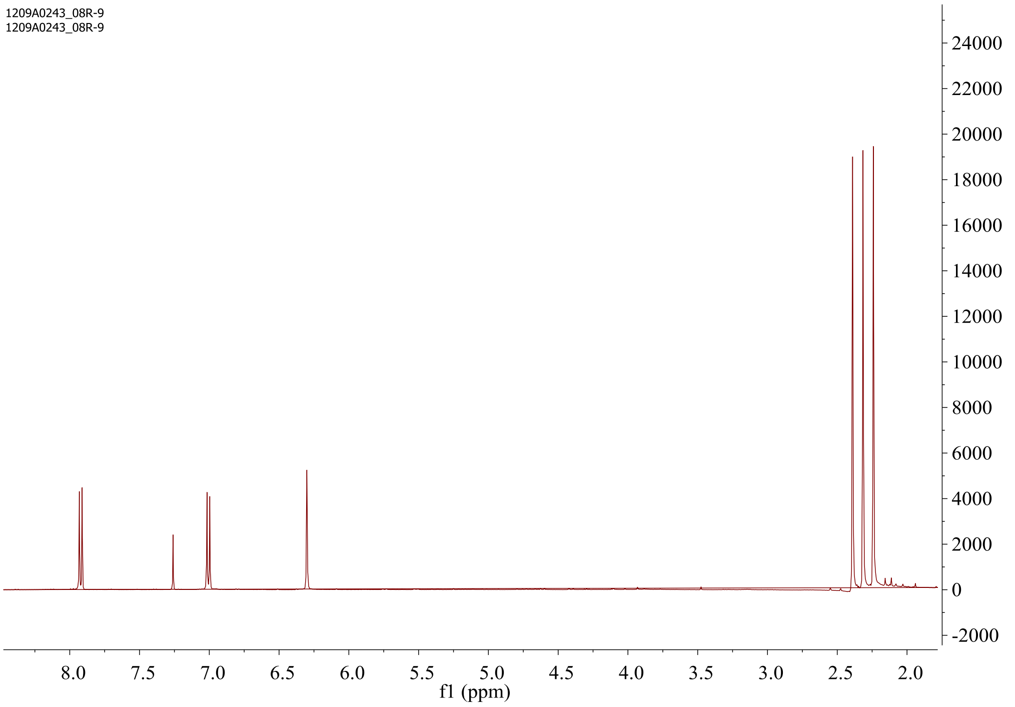

Figure S4 ^{1}H NMR spectrum of **1** in $CDCl_3$

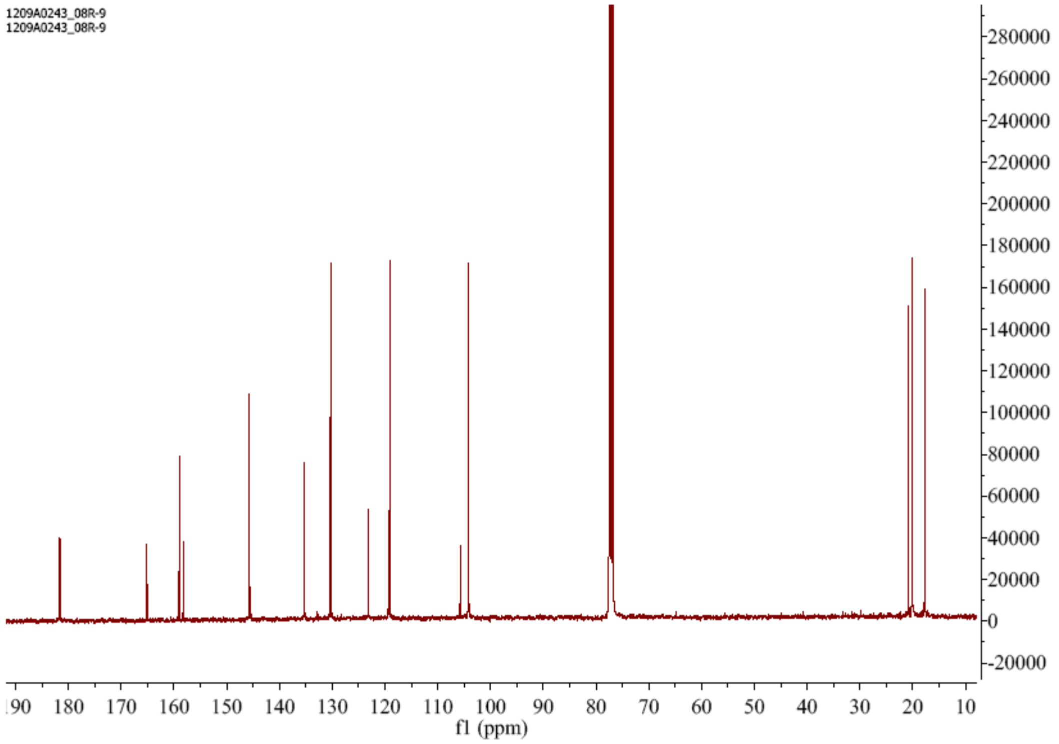

Figure S5 ^{13}C NMR spectrum of **1** in $CDCl_3$

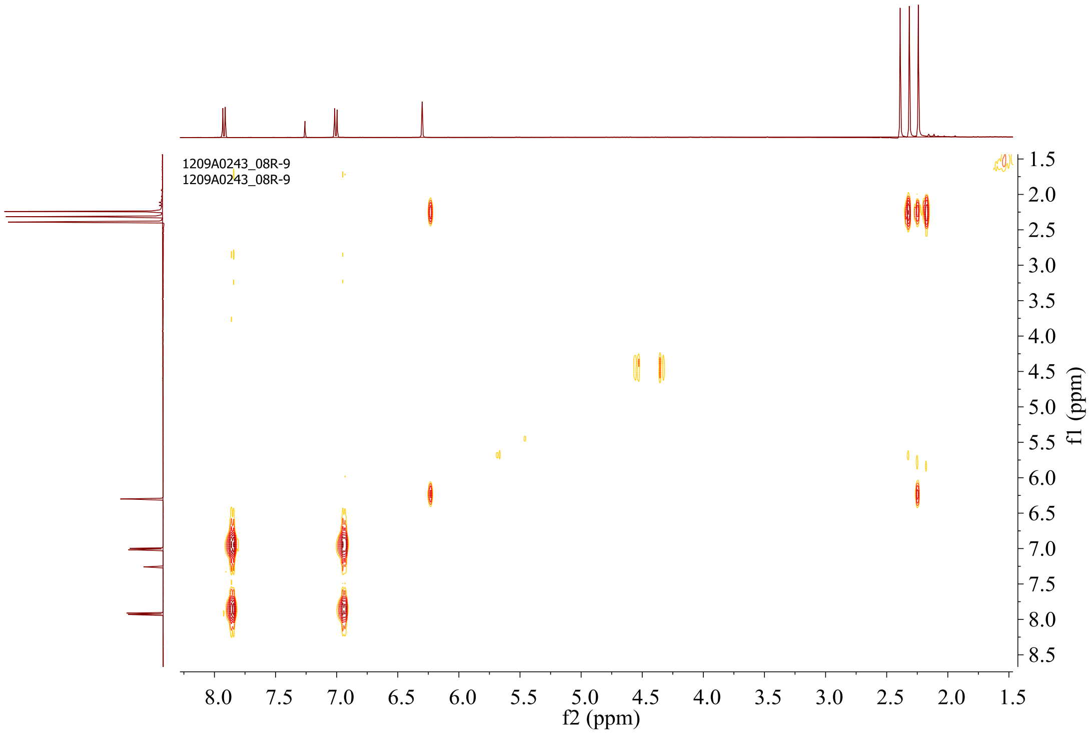

Figure S6 ^{1}H–^{1}H COSY spectrum of **1** in $CDCl_3$

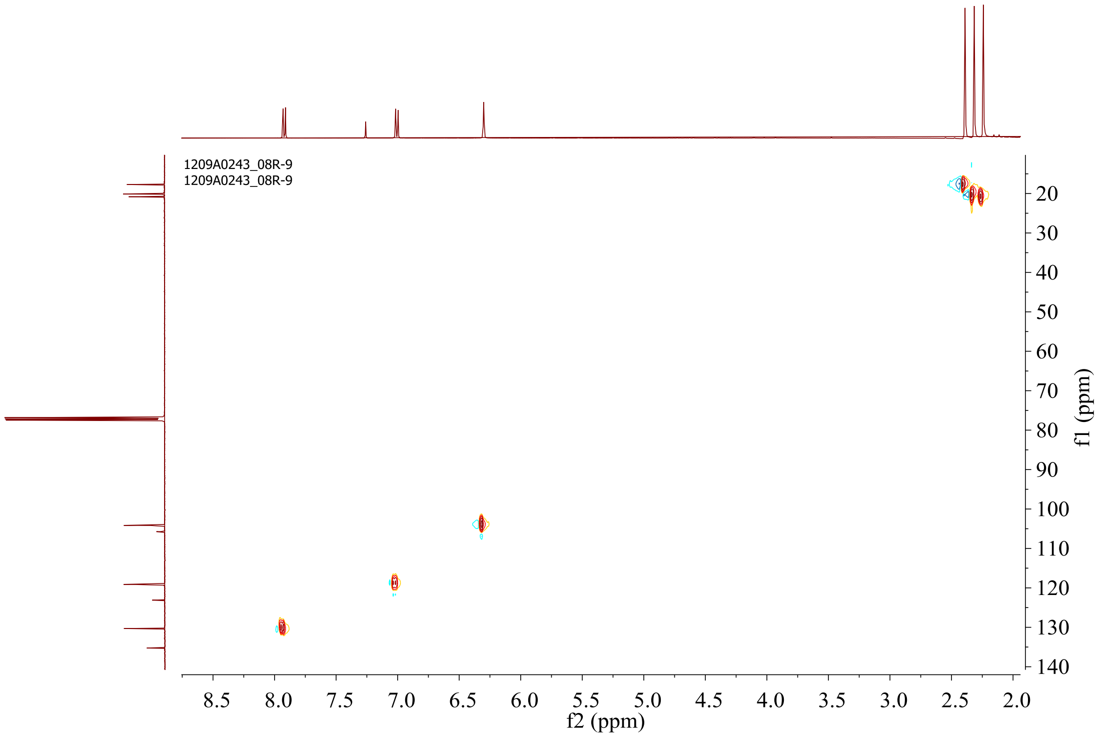

Figure S7 HSQC spectrum of **1** in $CDCl_3$

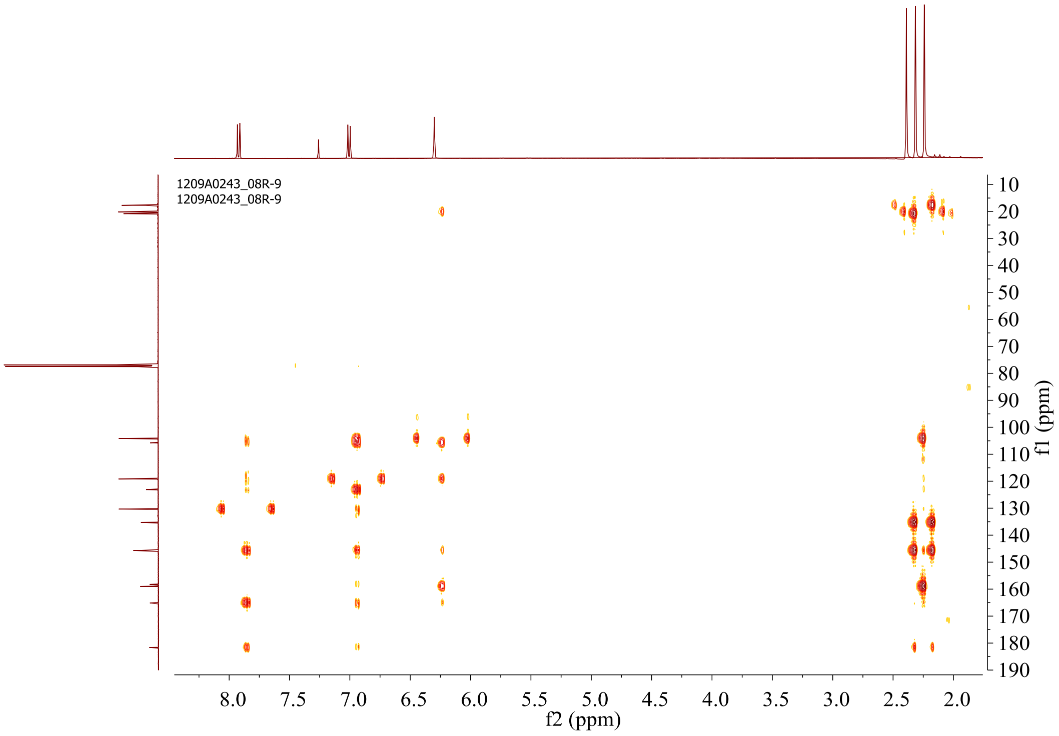

Figure S8 HMBC spectrum of **1** in $CDCl_3$

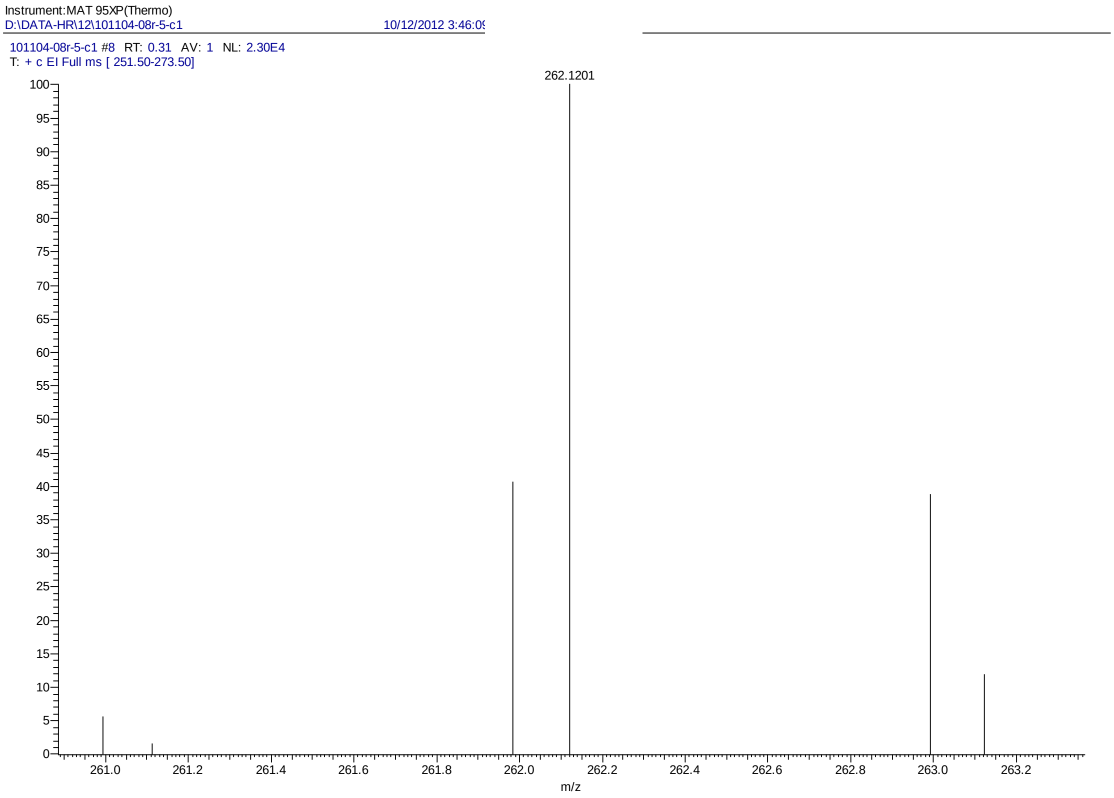

Figure S9 HREIMS spectrum of **2**

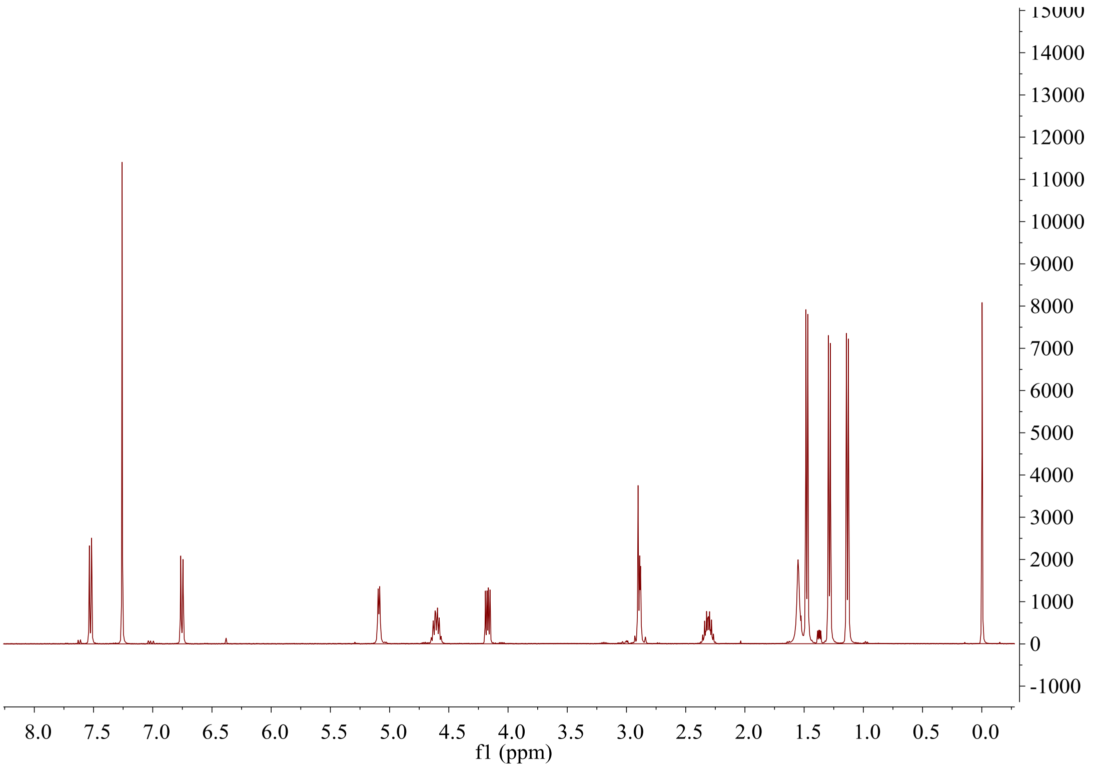

Figure S10 ^{1}H NMR spectrum of **2** in $CDCl_3$

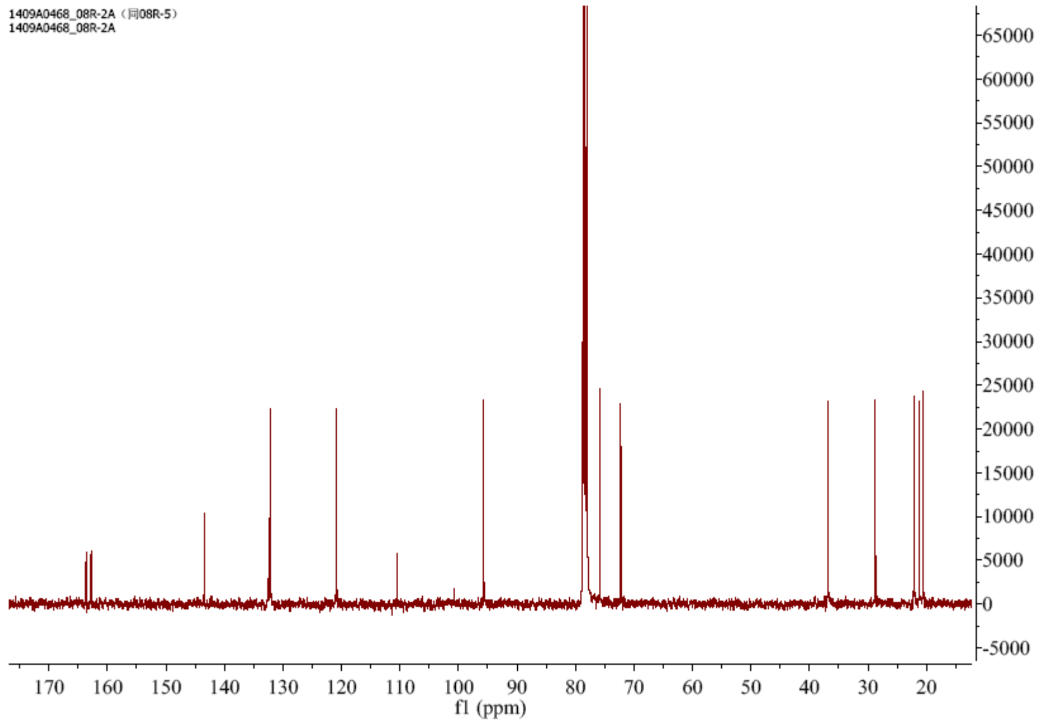

Figure S11 ^{13}C NMR spectrum of **2** in $CDCl_3$

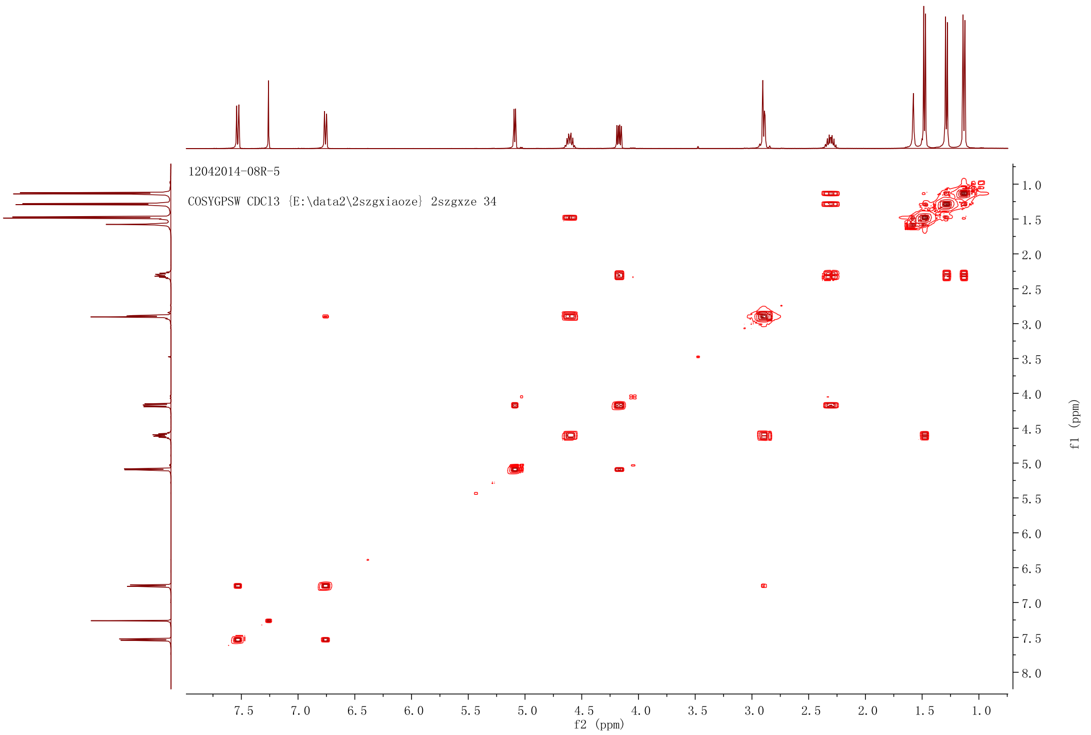

Figure S12 ^{1}H–^{1}H COSY spectrum of **2** in $CDCl_3$

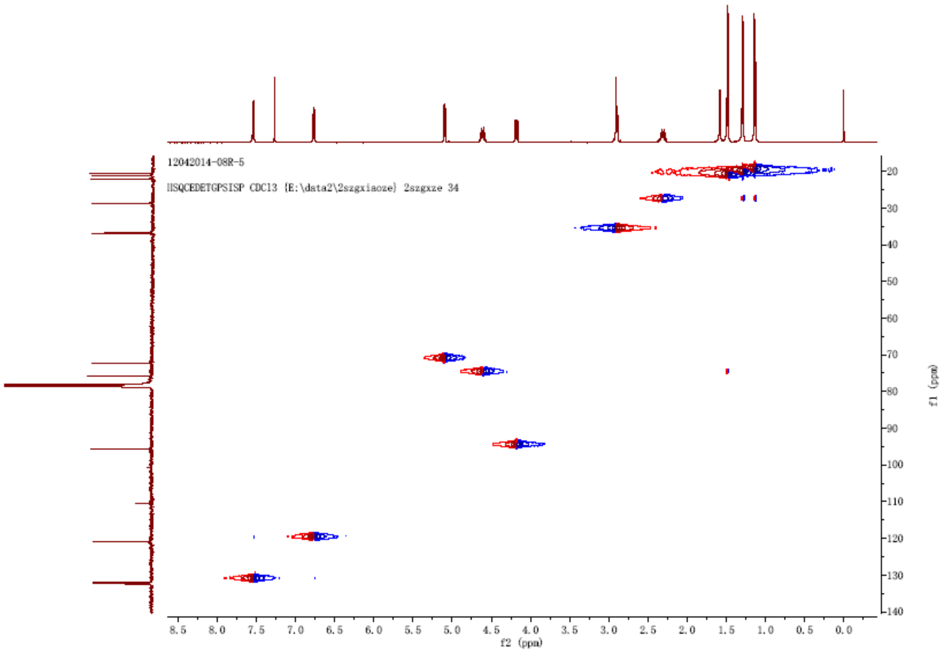

Figure S13 HSQC spectrum of **2** in $CDCl_3$

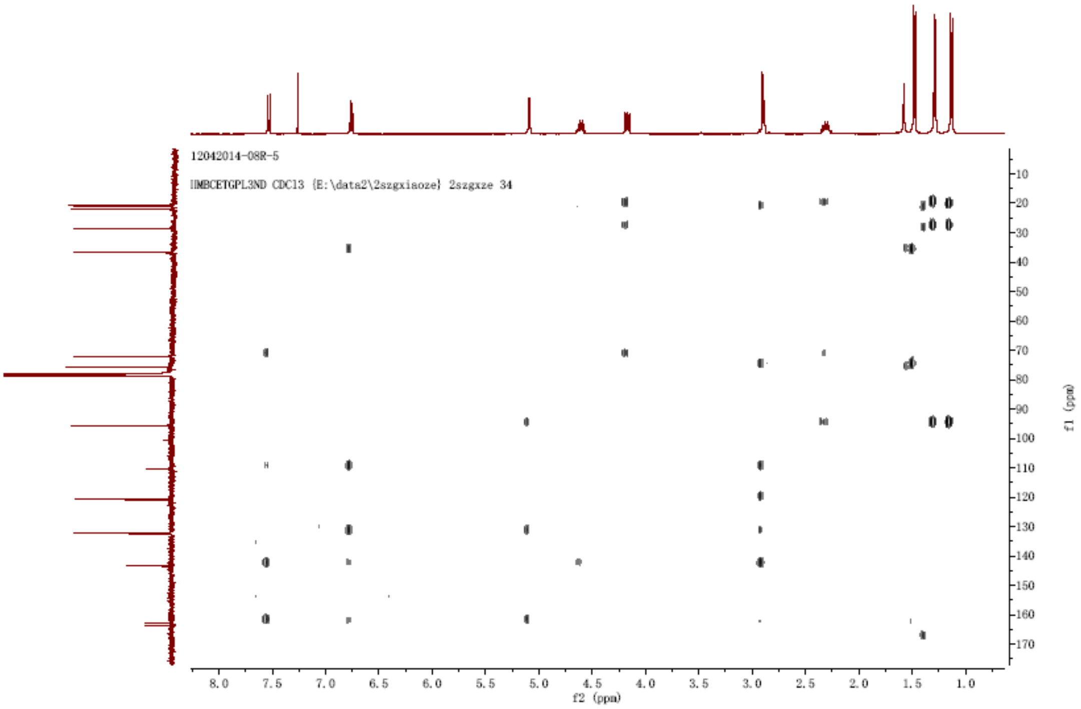

Figure S14 HMBC spectrum of **2** in $CDCl_3$

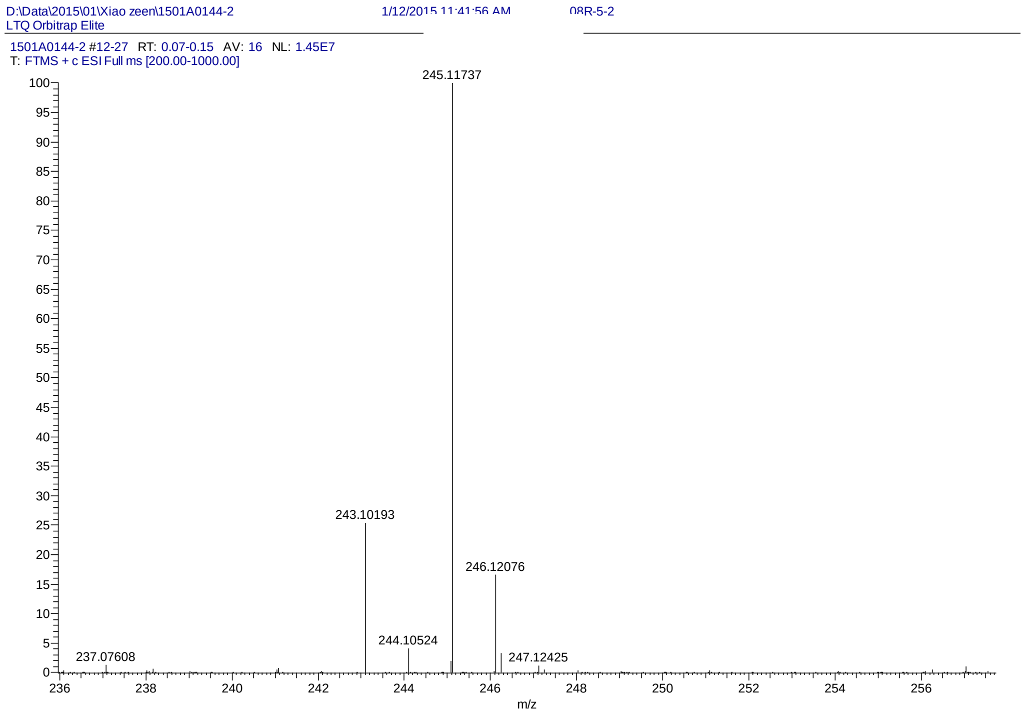

Figure S15 HRESIMS spectrum of **3**

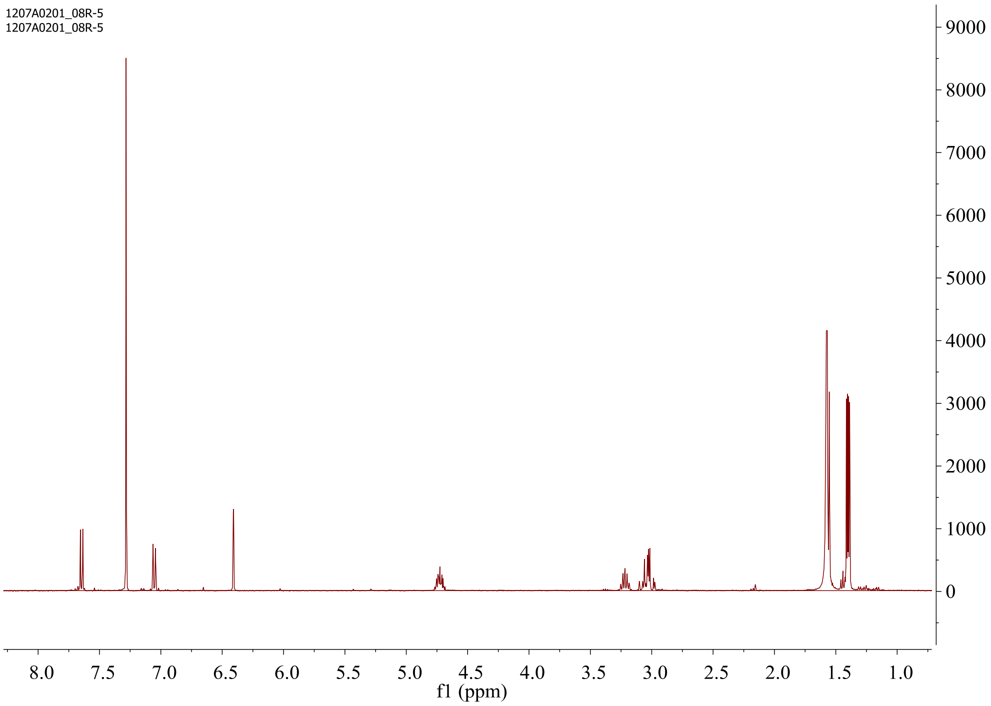

Figure S16 ^{1}H NMR spectrum of **3** in $CDCl_3$

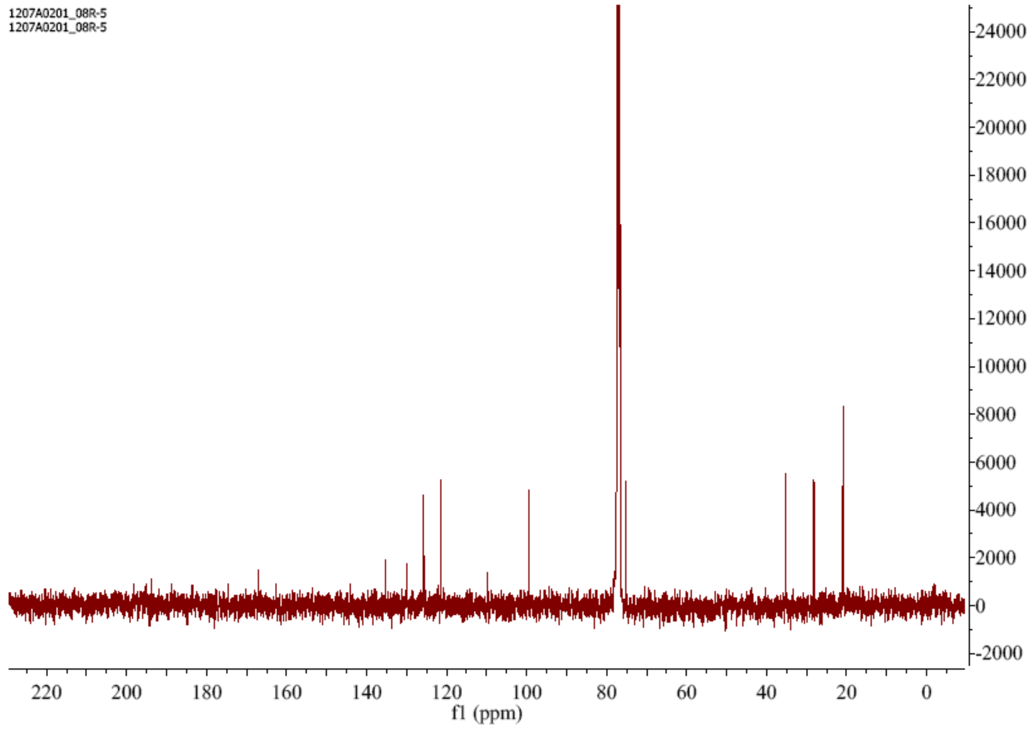

Figure S17 ^{13}C NMR spectrum of **3** in $CDCl_3$

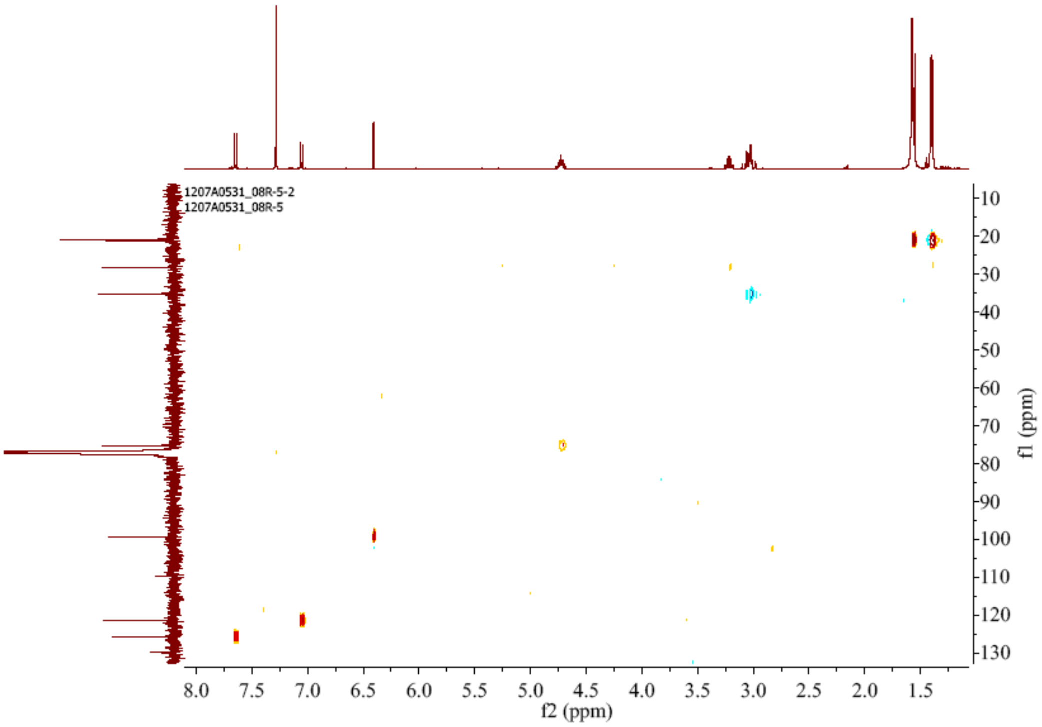

Figure S18 HSQC spectrum of **3** in $CDCl_3$

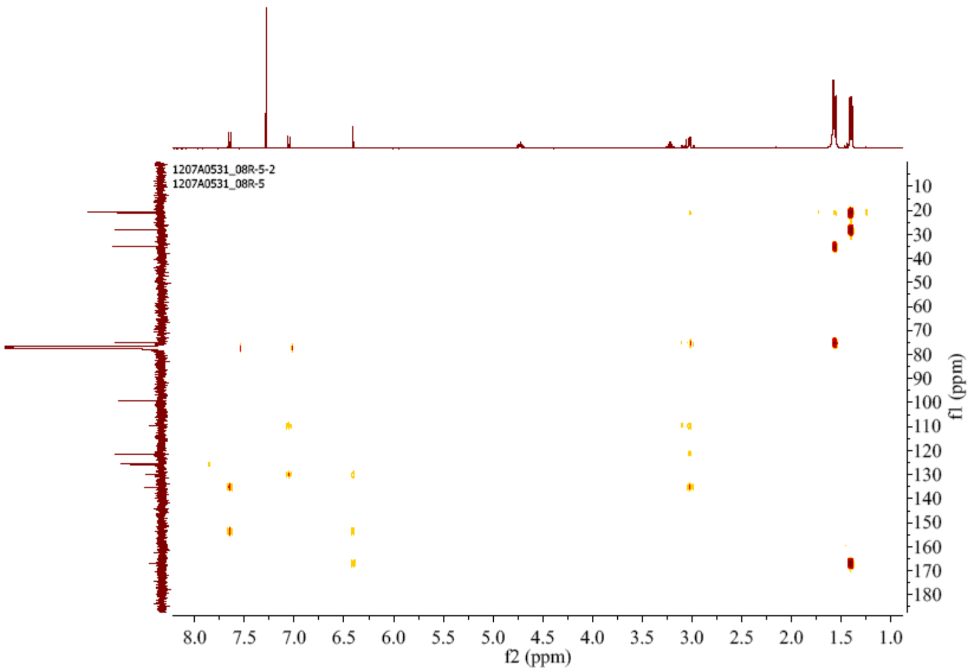

Figure S19 HMBC spectrum of **3** in $CDCl_3$

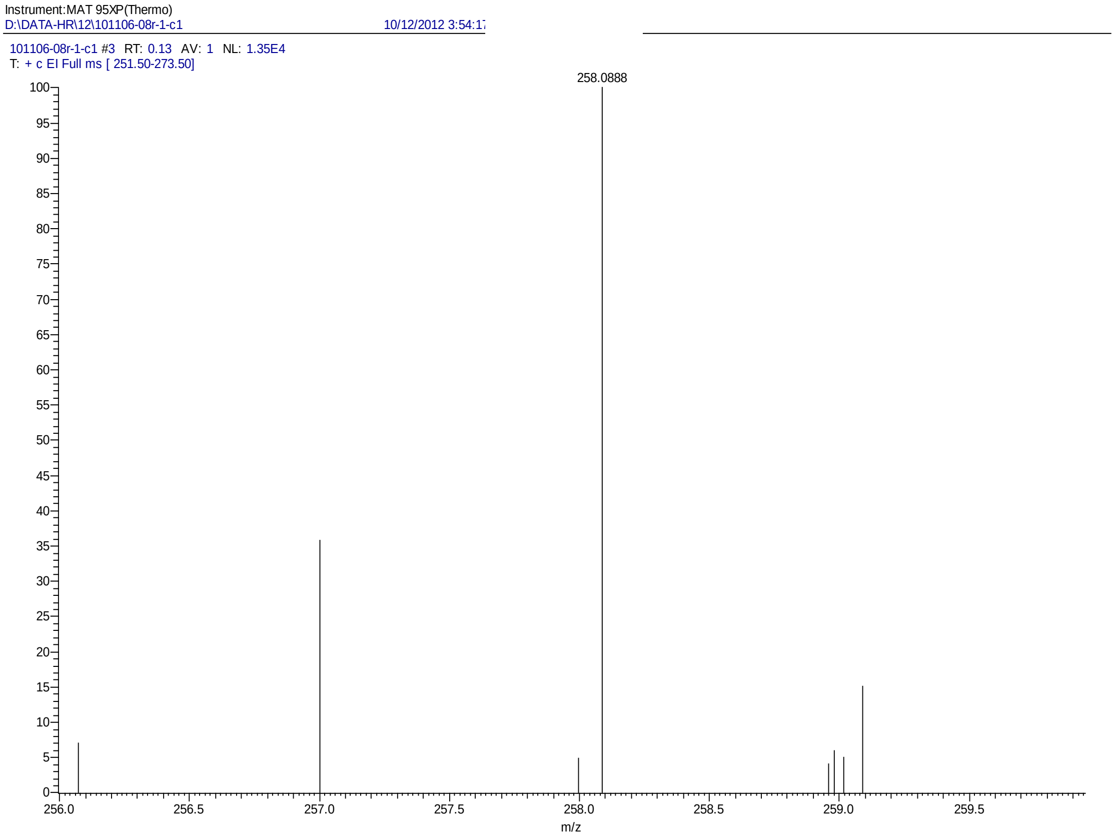

Figure S20 HRESIMS spectrum of **4**

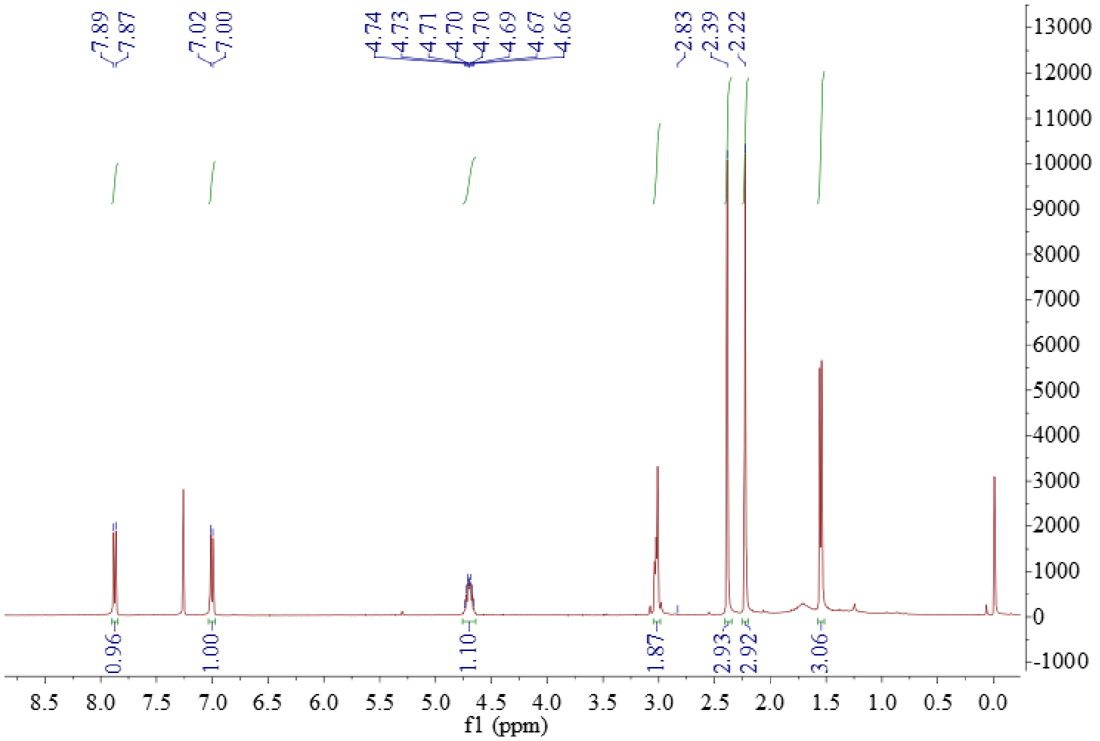

Figure S21 ^{1}H NMR spectrum of **4** in $CDCl_3$

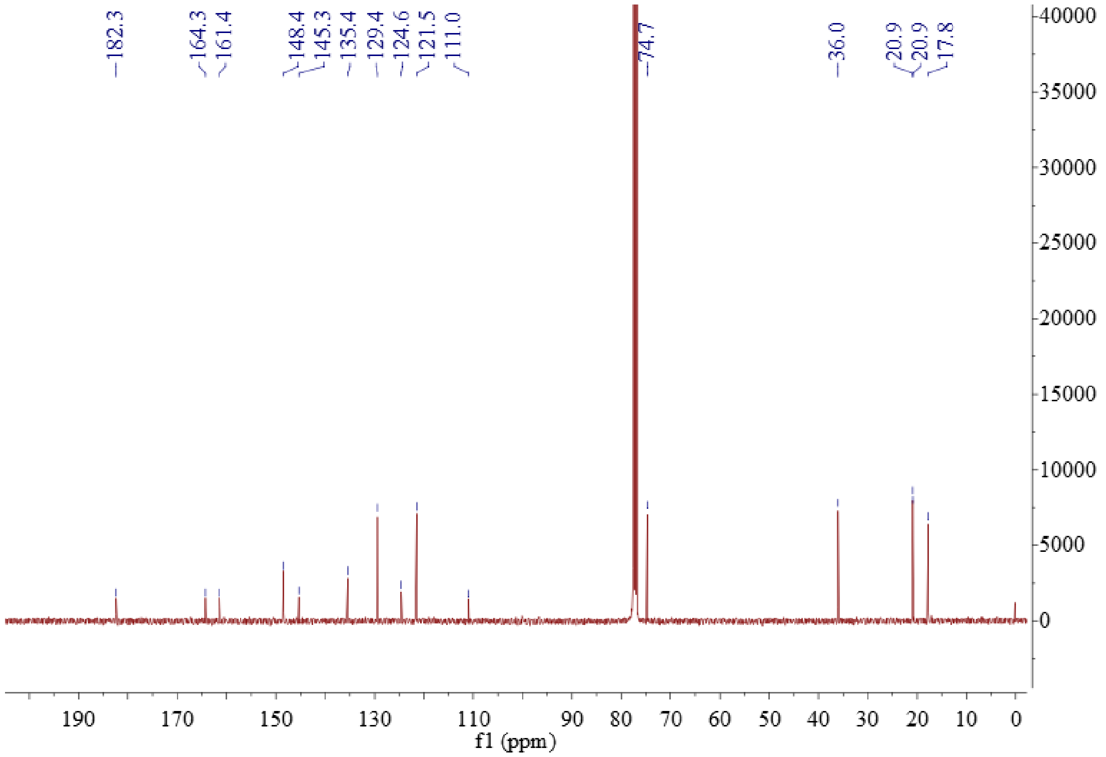

Figure S22 ^{13}C NMR spectrum of **4** in $CDCl_3$

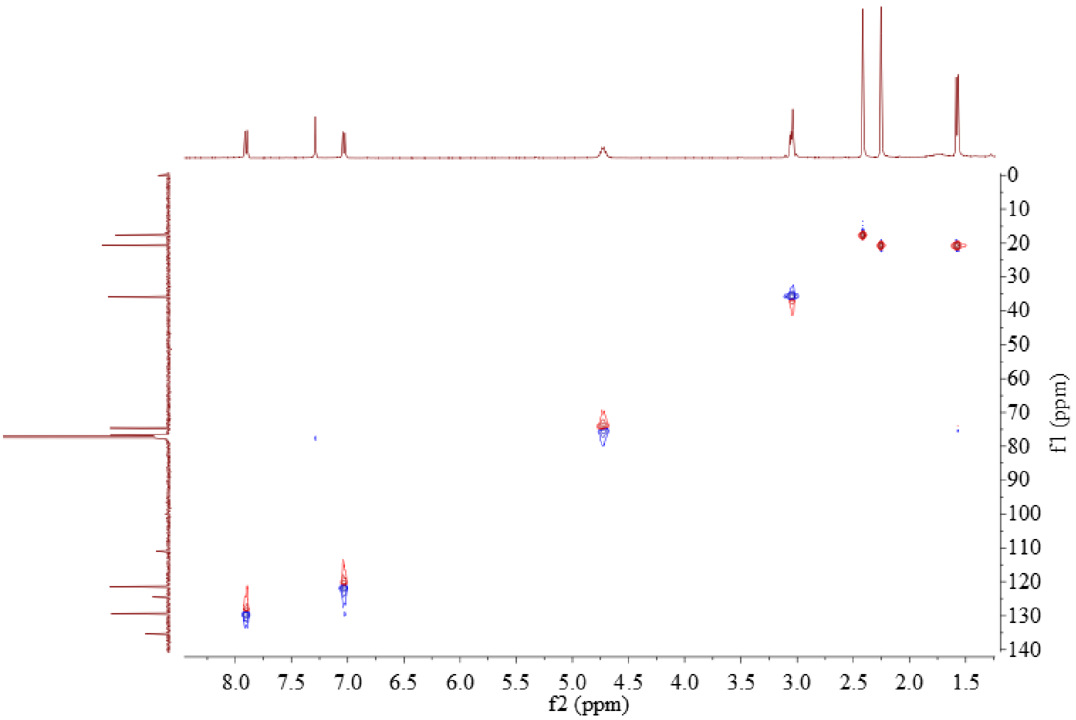

Figure S23 HSQC spectrum of **4** in $CDCl_3$

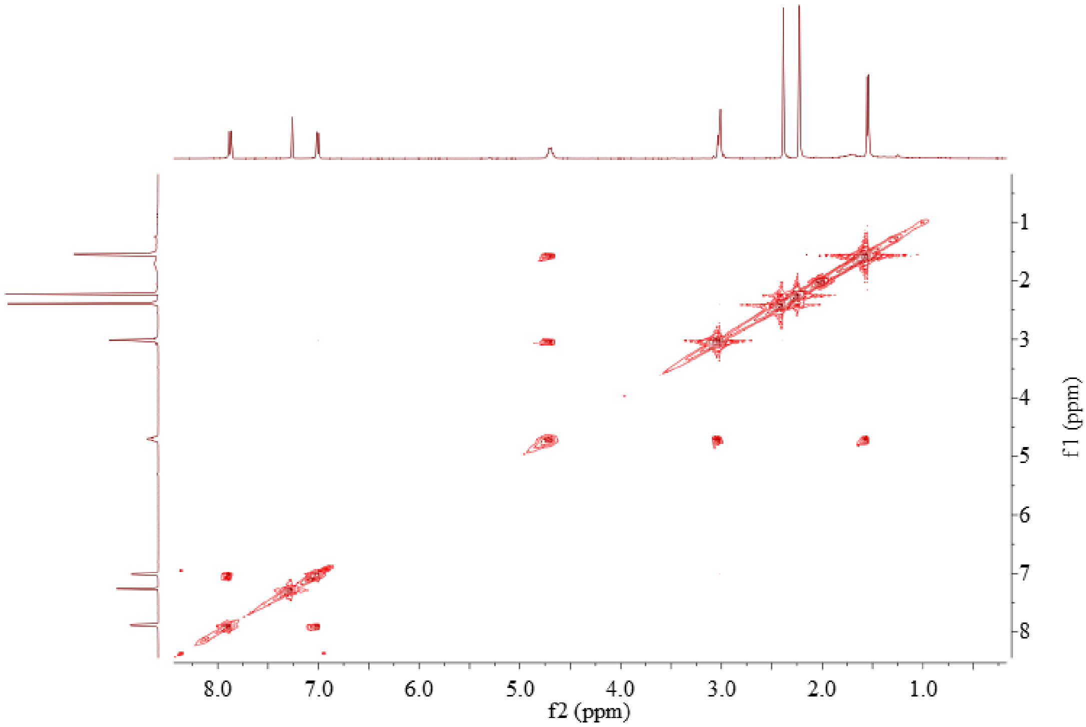

Figure S24 ^1H–^1H spectrum of **4** in $CDCl_3$

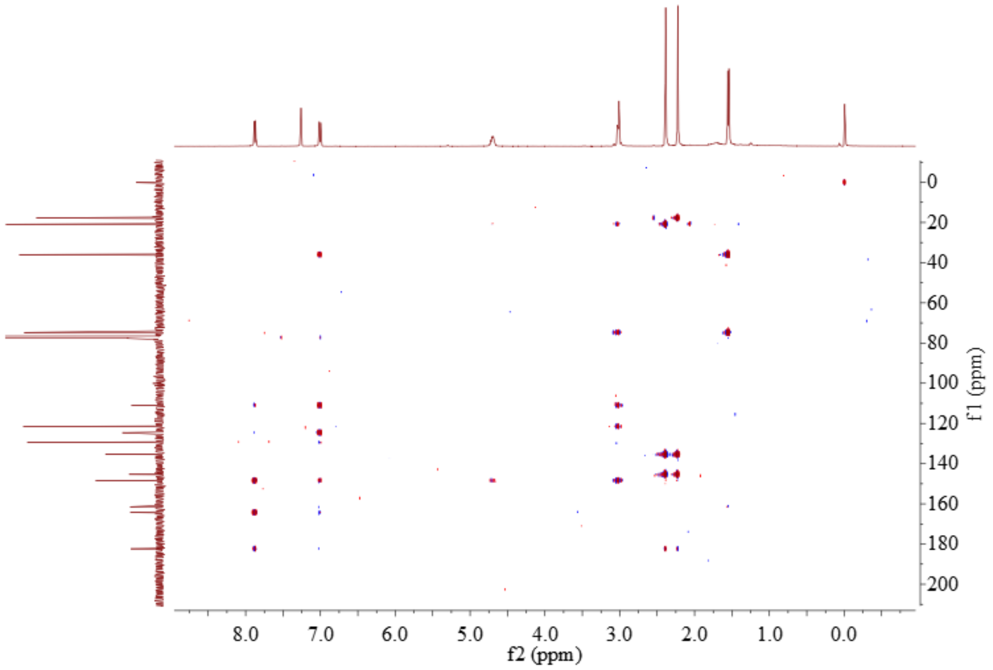

Figure S25 HMBC spectrum of **4** in $CDCl_3$

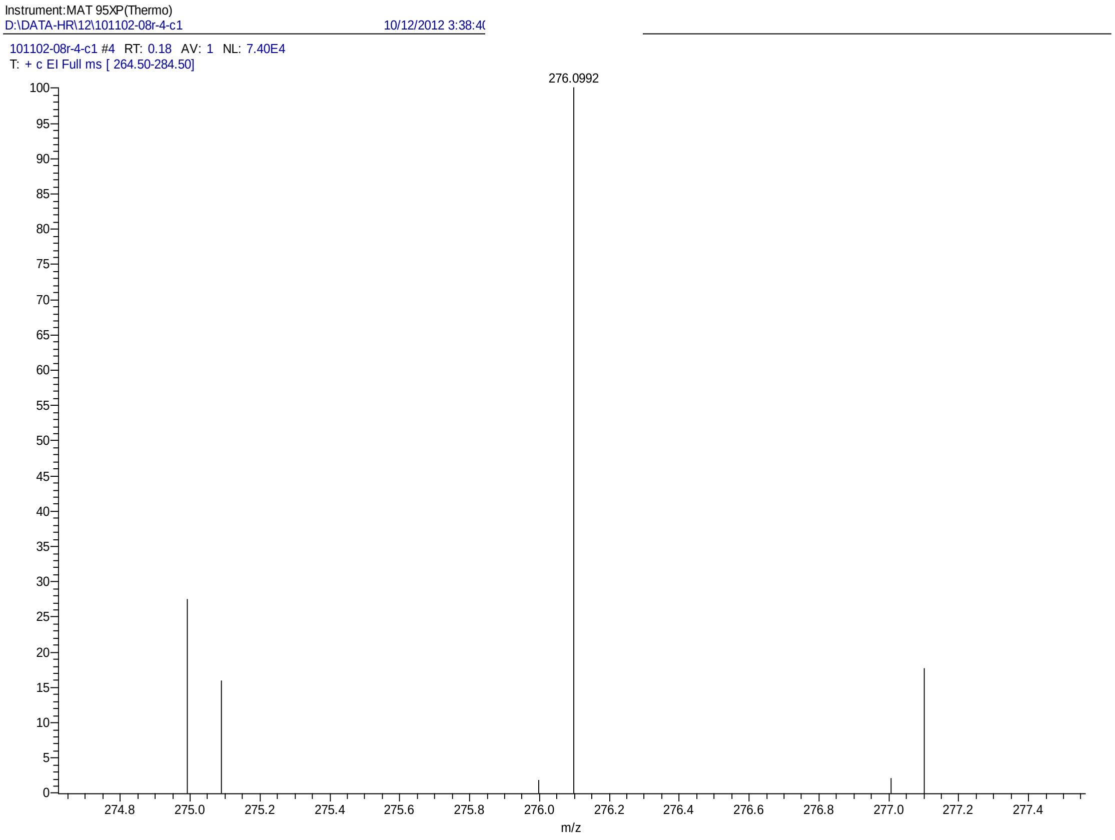

Figure S26 HREIMS spectrum of **5**

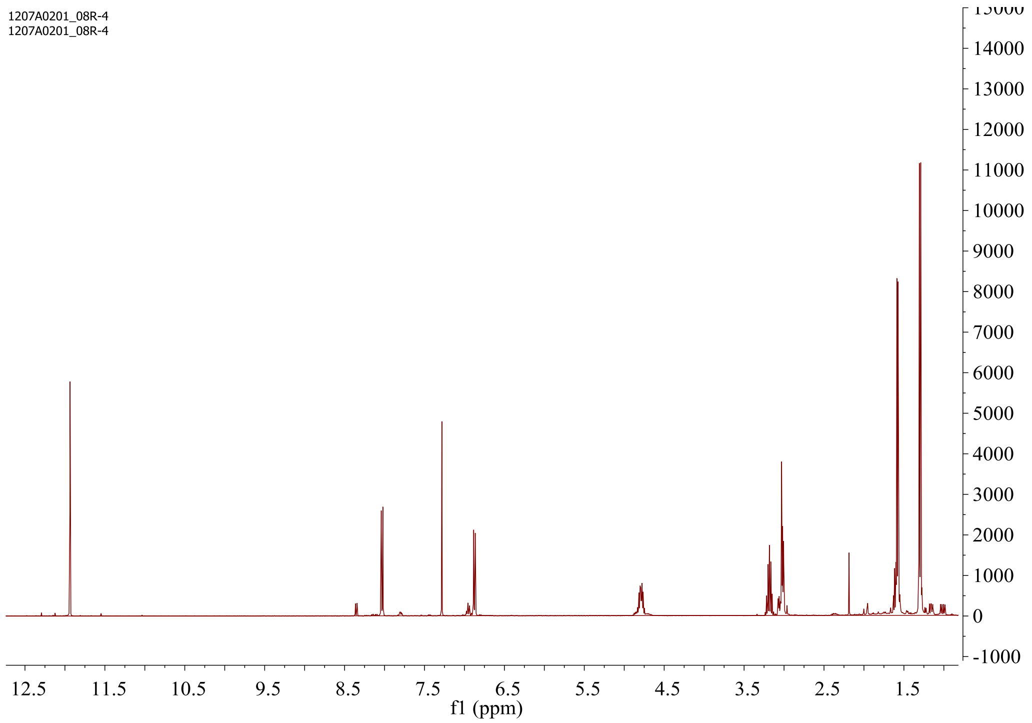

Figure S27 ^{1}H NMR spectrum of **5** in $CDCl_3$

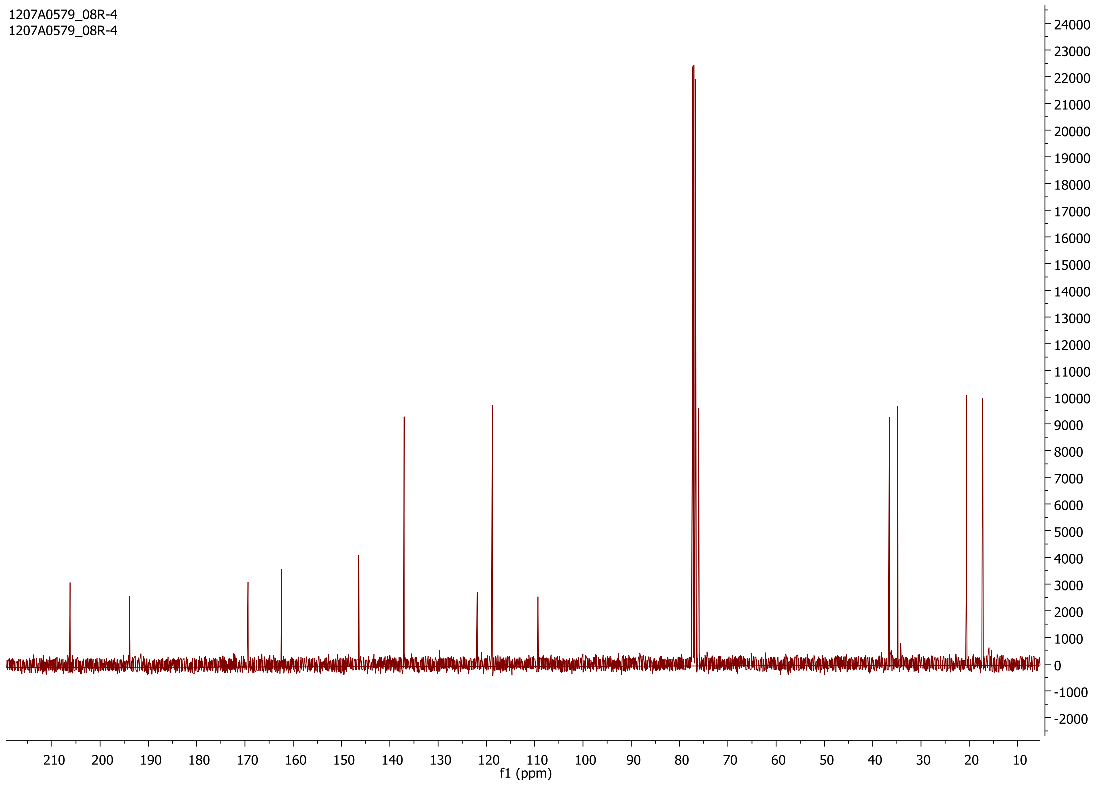

Figure S28 ^{13}C NMR spectrum of **5** in $CDCl_3$

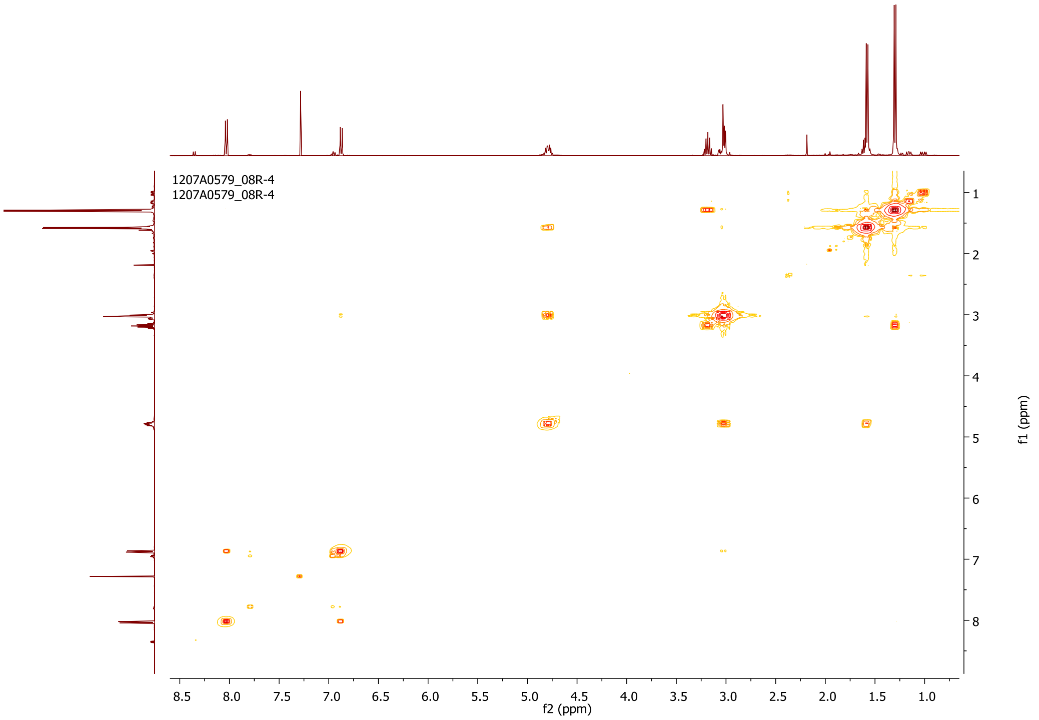

Figure S29 ^{1}H–^{1}H COSY spectrum of **5** in $CDCl_3$

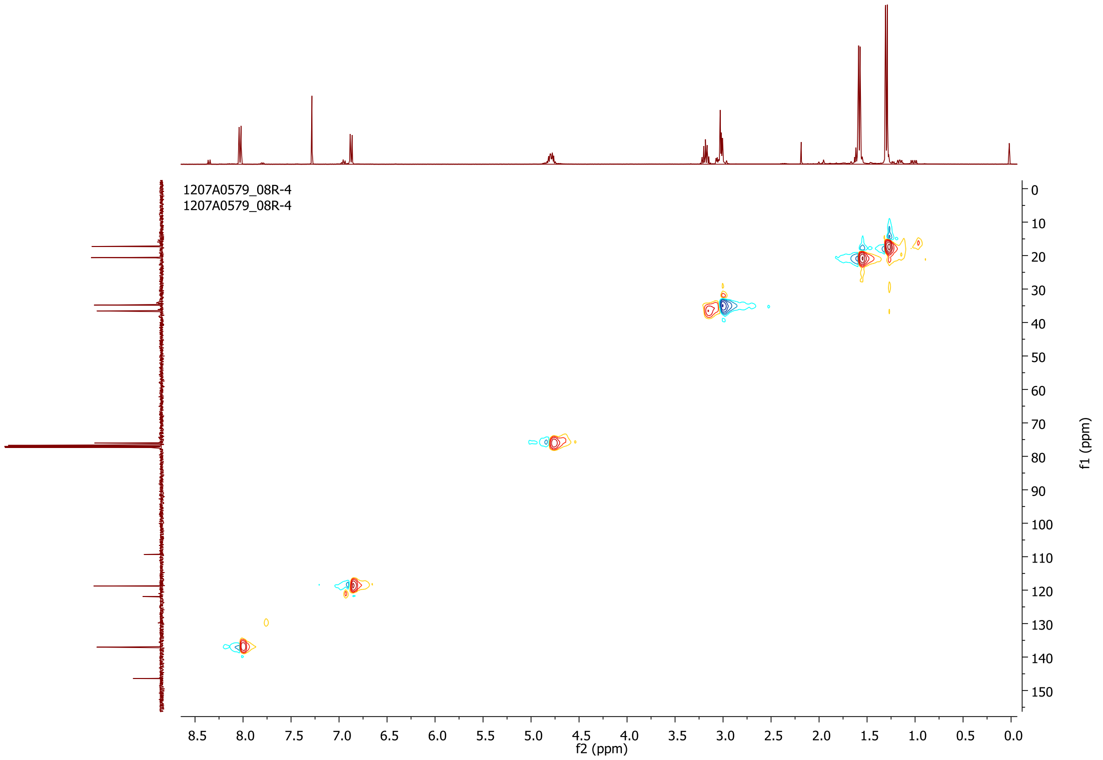

Figure S30 HSQC spectrum of **5** in $CDCl_3$

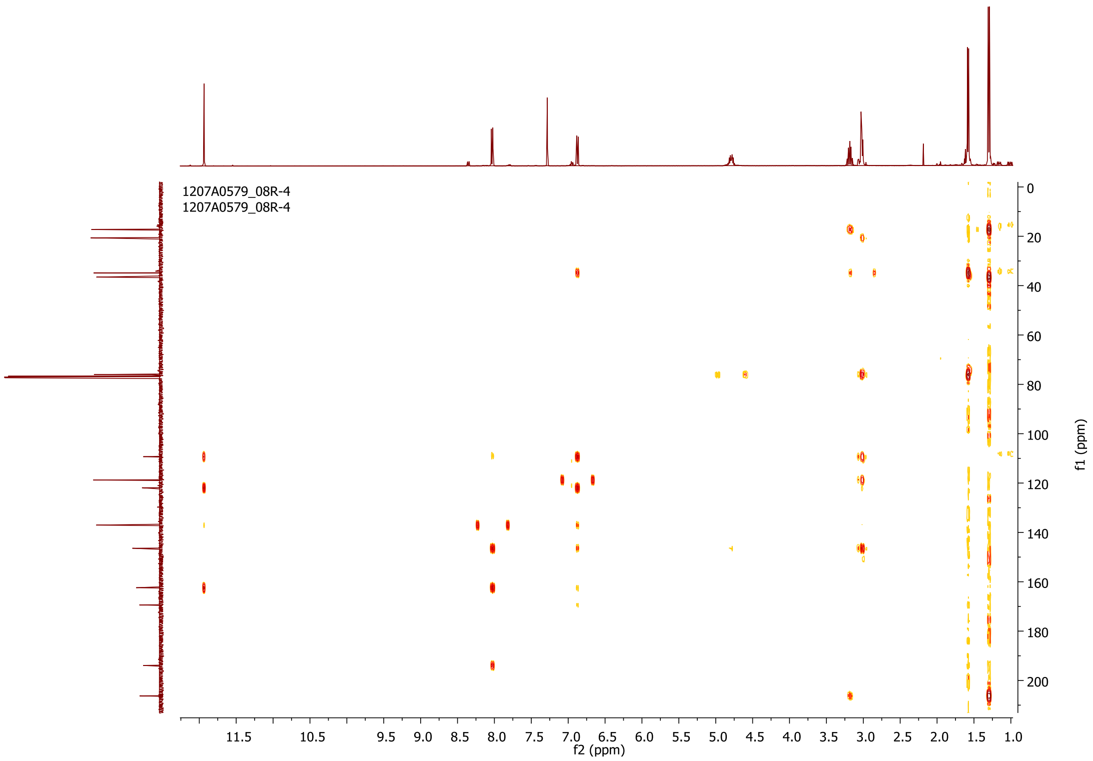

Figure S31 HMBC spectrum of **5** in $CDCl_3$

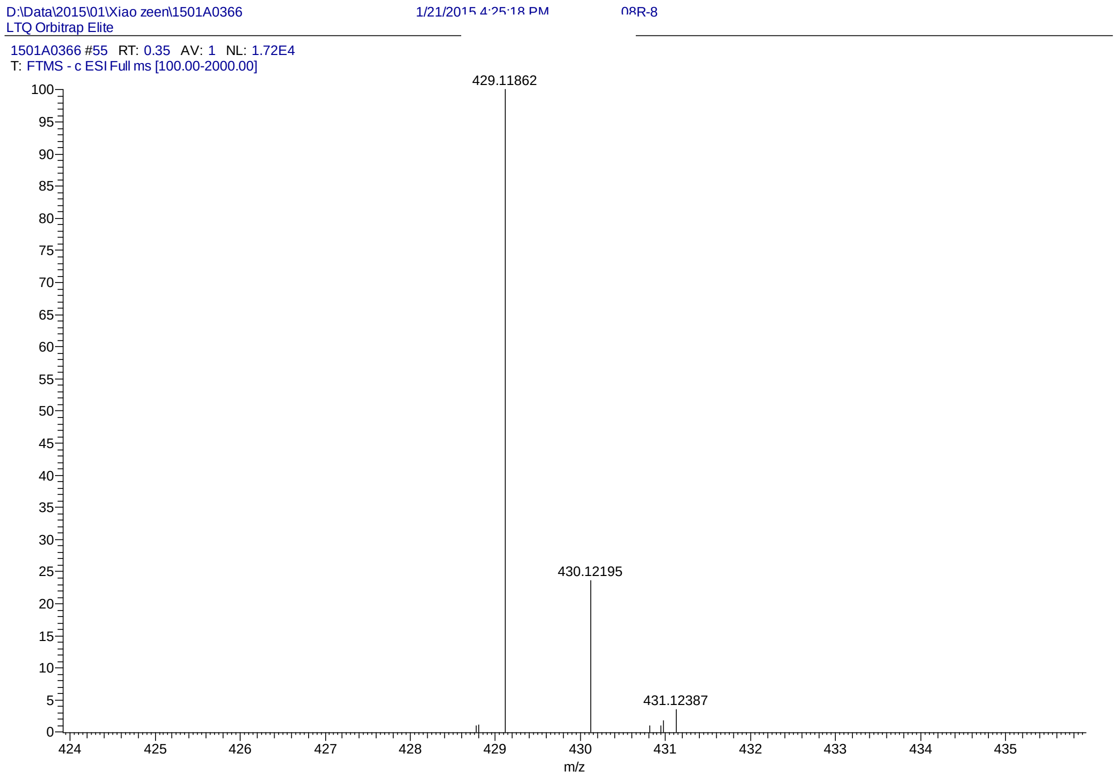

Figure S32 HRESIMS spectrum of **6**

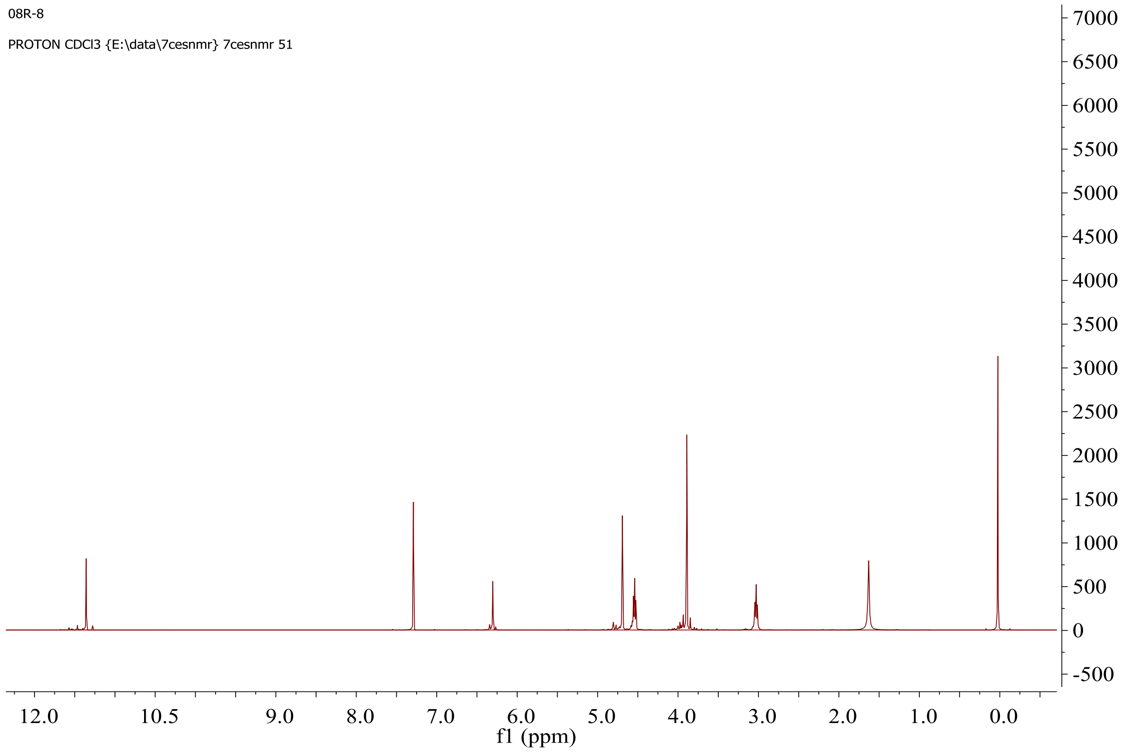

Figure S33 ^{1}H NMR spectrum of **6** in $CDCl_3$

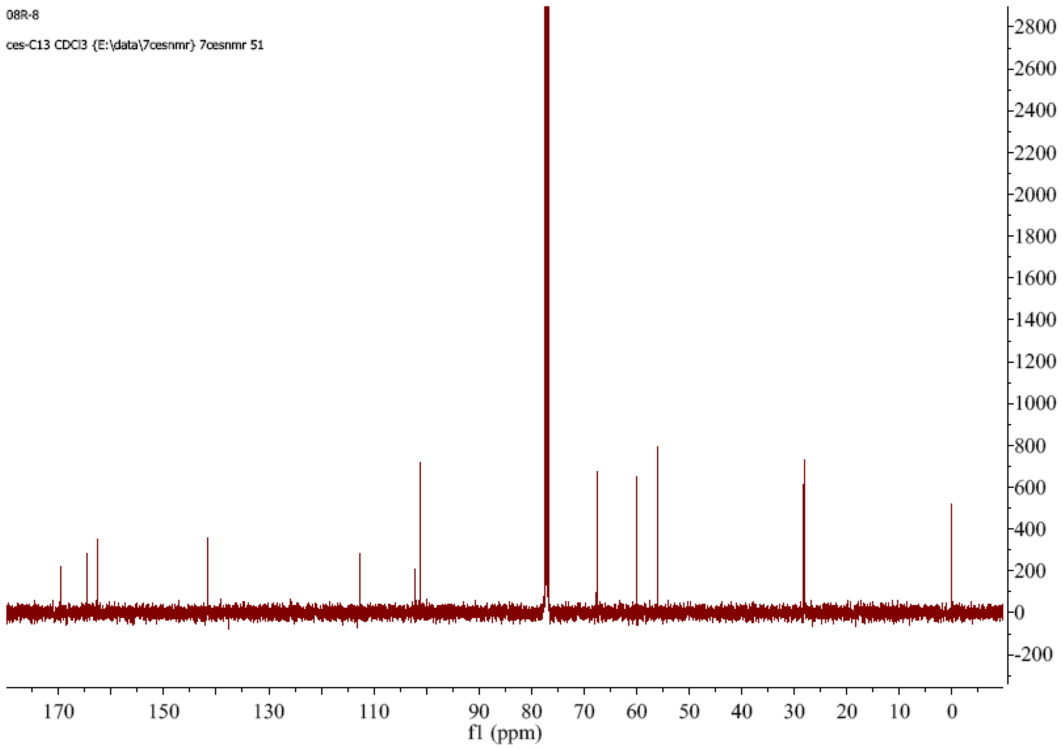

Figure S34 ^{13}C NMR spectrum of **6** in $CDCl_3$

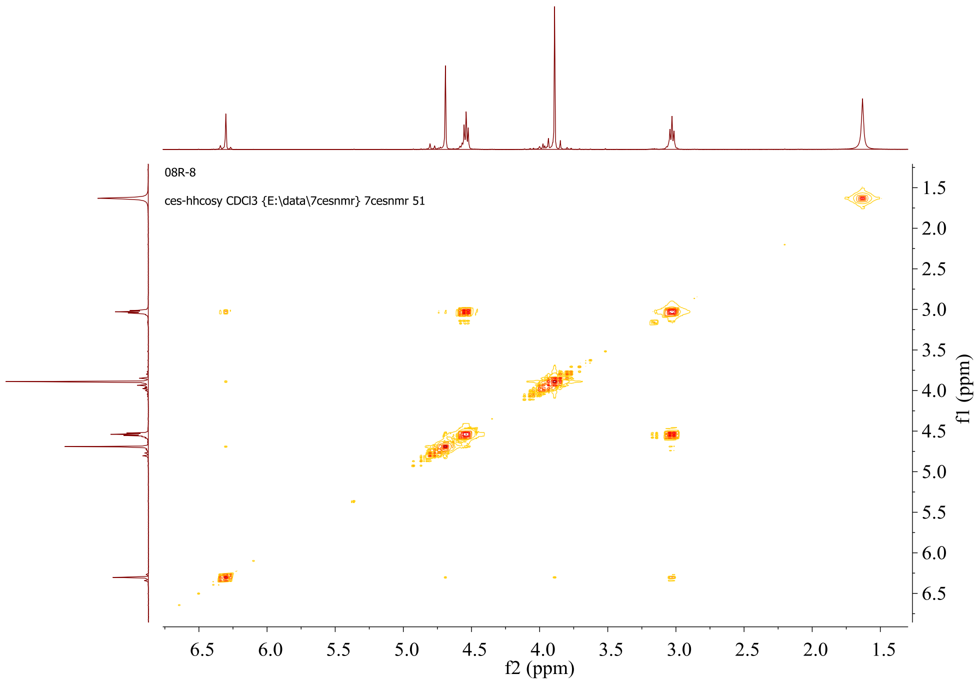

Figure S35 ^{1}H–^{1}H COSY spectrum of **6** in $CDCl_3$

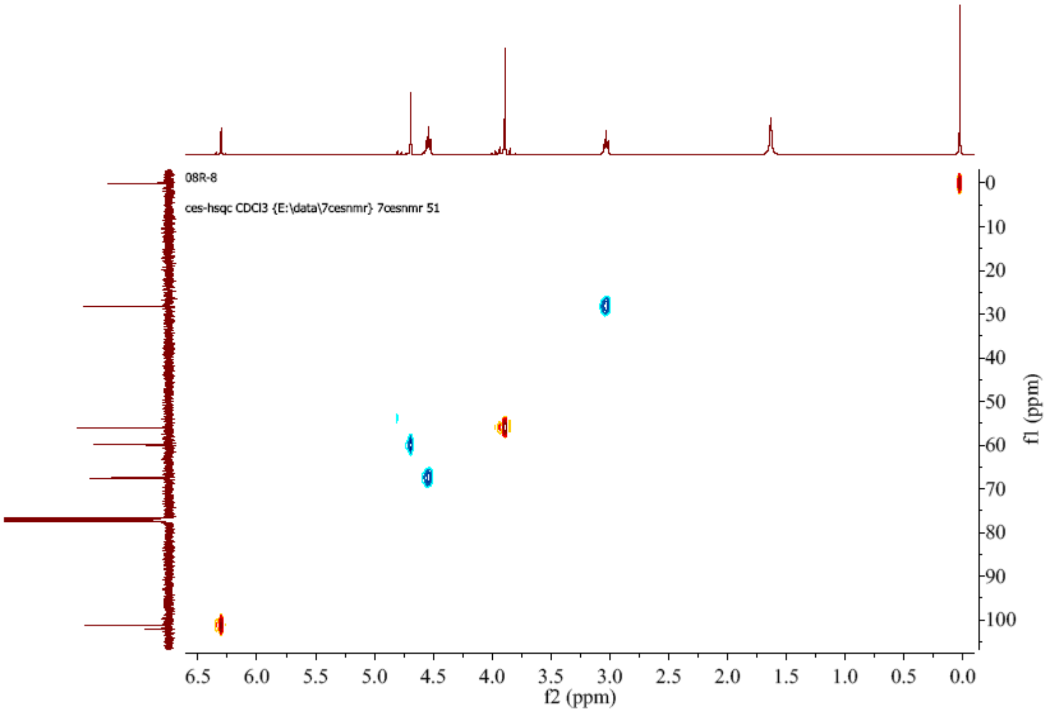

Figure S36 HSQC spectrum of **6** in $CDCl_3$

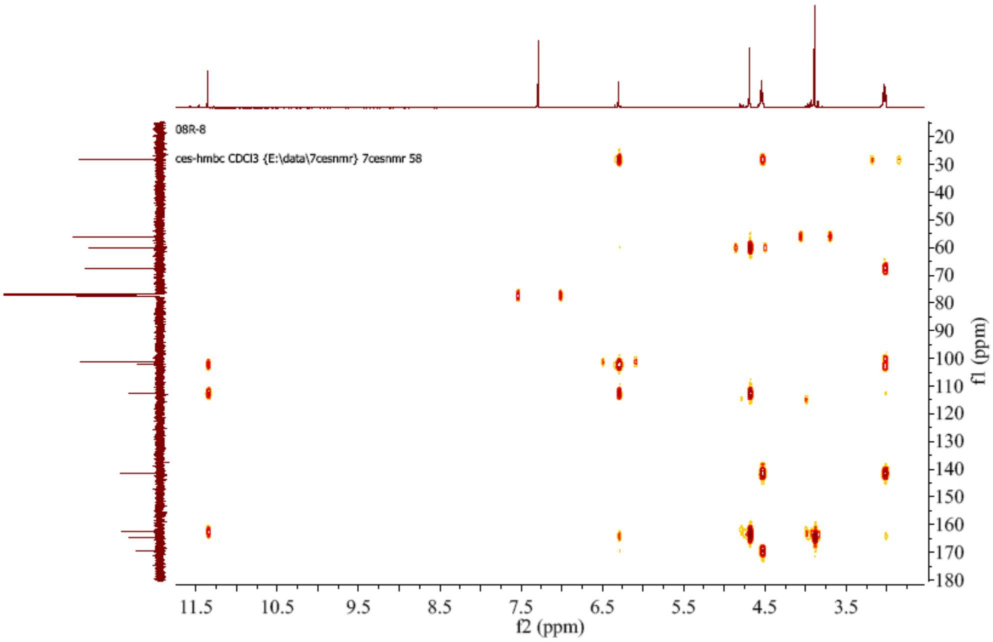

Figure S37 HMBC spectrum of **6** in $CDCl_3$
